# Acute stress in adulthood impoverishes social choices and triggers aggressiveness in preclinical models

**DOI:** 10.3389/fnbeh.2014.00447

**Published:** 2015-01-06

**Authors:** Anne Nosjean, Arnaud Cressant, Fabrice de Chaumont, Jean-Christophe Olivo-Marin, Frédéric Chauveau, Sylvie Granon

**Affiliations:** ^1^Centre de Neuroscience Paris Sud, Université Paris Sud 11 and Centre National de la Recherche Scientifique UMR 8195Orsay, France; ^2^Unité d'Analyse d'Images Quantitative, Institut Pasteur, Centre National de la Recherche Scientifique URA 2582Paris, France; ^3^Institut de Recherche Biomédicale des Armées, NCO, Unité NPSBrétigny-sur-Orge, France

**Keywords:** social interactions, decision, aggression, dominance, β2 nicotinic receptor, corticosterone

## Abstract

Adult C57BL/6J mice are known to exhibit high level of social flexibility while mice lacking the β2 subunit of nicotinic receptors (β2^−/−^ mice) present social rigidity. We asked ourselves what would be the consequences of a restraint acute stress (45 min) on social interactions in adult mice of both genotypes, hence the contribution of neuronal nicotinic receptors in this process. We therefore dissected social interaction complexity of stressed and not stressed dyads of mice in a social interaction task. We also measured plasma corticosterone levels in our experimental conditions. We showed that a single stress exposure occurring in adulthood reduced and disorganized social interaction complexity in both C57BL/6J and β2^−/−^ mice. These stress-induced maladaptive social interactions involved alteration of distinct social categories and strategies in both genotypes, suggesting a dissociable impact of stress depending on the functioning of the cholinergic nicotinic system. In both genotypes, social behaviors under stress were coupled to aggressive reactions with no plasma corticosterone changes. Thus, aggressiveness appeared a general response independent of nicotinic function. We demonstrate here that a single stress exposure occurring in adulthood is sufficient to impoverish social interactions: stress impaired social flexibility in C57BL/6J mice whereas it reinforced β2^−/−^ mice behavioral rigidity.

## Introduction

Social interactions involve highly integrative and adapted behaviors to make coherent decisions in specific environmental contexts. Social interactions are altered in numerous psychiatric pathologies, such as anxiety (Lukkes et al., 2009; Morrison and Heimberg, 2013), depression (Anisman and Matheson, 2005), schizophrenia (Marwick and Hall, 2008; Ritsner et al., 2013), drug abuse (Buckner et al., 2013), autism (Grzadzinski et al., 2013; Zafeiriou et al., 2013), post-traumatic stress disorders (Javidi and Yadollahie, 2012; Zoladz and Diamond, 2013), and under stressful conditions (Lupien et al., 2009; Del Giudice et al., 2011; Javidi and Yadollahie, 2012; Starcke and Brand, 2012). Social interactions require sequential, and sometimes competing, choices that integrate both physiological and psychological parameters that are peculiar to the individual. Among these parameters, those that affect emotional, motivational, and memory processes are crucial (Adolphs, 2010; Gasbarri and Tomaz, 2012; Adolphs and Anderson, 2013).

The prevalence of stress-induced pathologies has increased tremendously in industrialized countries, to the extent that they have become a major global health problem. An individual's ability to prevent, correct, or escape stressful situations to reach a state of personal well-being reflects the overall health of society. Numerous studies have addressed the short- and long-term effects of chronic stress on brain physiology and plasticity, on cognitive function in adults, and on cognitive function during childhood and adolescence (Veenema, 2009; Marquez et al., 2013); other studies have focused on the social impact of chronic stress and the triggering of aggressiveness (Sandi et al., 2008; McEwen, 2012; Barik et al., 2013). Aggressive behavior is common and adaptive between rodents of the same species and, like all mammals, they cope with social threats by making physiological and behavioral adjustments that can increase their ability to escape or fight. The stress-response system and the activation of the hypothalamus-pituitary-adrenal axis (HPA) axis are known to support these adjustments (for reviews, Koolhaas et al., 1999; Nevo, 2007). In laboratory, acute stress has also been intensely studied, particularly its impact on memory (for reviews, Roozendaal, 2002, 2003; McEwen, 2006, 2007; Sandi and Pinelo-Nava, 2007; Roozendaal et al., 2009; Van Der Kooij and Sandi, 2012). It has also been shown to affect decision-making in humans (Porcelli and Delgado, 2009; Starcke and Brand, 2012; Pabst et al., 2013a,b) and in rodents (Quartermain et al., 1996; Toledo-Rodriguez and Sandi, 2011; Shafiei et al., 2012; Butts et al., 2013). Surprisingly, however, the consequences of acute stress on the complexity of social interactions have received little attention to date, despite the fact that, in people's minds, it is widely believed that acute stress can produce disproportionate and potentially disastrous reactions in social circumstances (but see a recent study in humans by Von Dawans et al., 2012). It is known that stress alters the function of the prefrontal cortex (PFC), a brain structure implicated in the social cognition and not fully developed before adulthood (Marquez et al., 2013). However, the effect of acute stress in adults, i.e., when the brain is fully developed, on social interaction has never been clearly established. We thus intended to address this question here.

Mice that lacked the β2 subunit of nicotinic receptors (β2^−/−^ mice) did not exhibit cognitive defects (Zoli et al., 1999; Wiklund et al., 2009) and showed normal levels of motivation in response to natural rewards (Chabout et al., 2013); however, they presented less flexible behaviors i.e., exhibited behavioral rigidity when motivations were in conflicting (Granon et al., 2003; de Chaumont et al., 2012). We recently evidenced that the role of neuronal nicotinic receptors -nAChRs- depends on the degree of safety or uncertainty of a behavioral context: in a secured social context, β2-nAChRs are not necessary for integrating social information or social rewards, whereas in uncertain context they are essential for the management and the hierarchical organization of choices (Avale et al., 2011; Serreau et al., 2011; Chabout et al., 2013). Functional nAChRs and efficient cholinergic neurotransmission are thus needed for adapted social skills and other executive functions (Granon et al., 2003; Kobayashi et al., 2013) as well as for the control of emotions and mood either in animals (Anderson and Brunzell, 2012; Mineur et al., 2013) or in humans (Tsai et al., 2012). In addition, an interesting study evidenced the effect of prenatal stress on the upregulation of nAChRs at adulthood (Schultz et al., 2013). Together, the data suggest a direct role of nAChRs in social skills as well as in stress management but the contribution of the various nicotinic receptors subunits, in particular that of the β2 subunit in these processes, is not defined. We expected here to provide novel information to this regard.

Social behaviors are complex behaviors that require sequential choices to achieve an action and integrate both emotional and motivational processes (Adolphs, 2010; Starcke and Brand, 2012). In our laboratory, we currently used a social interaction task in which a previously isolated adult mouse, e.g., deprived of social contact for 3–4 weeks, makes continuous free choices between exploration of a novel environment and social contacts with an unknown social-housed mouse of same sex and age (Granon et al., 2003; Avale et al., 2011; Serreau et al., 2011; de Chaumont et al., 2012). Such isolation time was chosen in order to reinforce the rewarding value of social contacts (Granon et al., 2003; Krach et al., 2010) since non-isolated animals were not highly interested in social interaction (Avale et al., 2011; Chabout et al., 2013). Similar isolation procedures during adulthood, largely used in many learning paradigms, seem to be unsufficient alone to induce chronic stress (Sakakibara et al., 2012). Furthermore, in our social interaction task, the isolated animals explored the novel environment before the task. If not, the amount of time spent in social contact dropped around 15 percent (Avale et al., 2011). Under these experimental conditions, C57BL/6J mice, which are known to be highly sociable (Sankoorikal et al., 2006), made frequent social contacts; exhibited high levels of exploration (Avale et al., 2011), a normal level of dominant behaviors, and no aggressive behaviors (Coura et al., 2013). In contrast, adult β2^−/−^ mice favored social contacts to the detriment of the exploration and lacked behavioral flexibility and adaptation of ongoing behavior to environmental changes (for review, Kehagia et al., 2010). They also showed more dominant behaviors than C57BL/6J mice (Coura et al., 2013). Since we established that β2^−/−^ mice lacked behavioral flexibility and exhibit more rigid choices in social or in non-social choice tasks, we hypothesized that they would be more sensitive to stress and as such, we wondered whether stress could worsen or prevent β2^−/−^ mice social rigidity.

In a recent work, we validated a software highlighting social behaviors (MiceProfiler, de Chaumont et al., 2012). In particular we investigated in detail the difference between the analyses conducted by two independent investigators from that using the software. We showed that some events were similarly scored by manual and automatic analyses (e.g., contacts) while some were under evaluated by manual scoring (follows) or simply undetectable by visual analysis (complex events). In the aforementioned study, we only focused on some remarkable social events occurring during the task. Here, we further investigated each behavioral event of each mice of the dyad, i.e., contact and single events, postures, dynamic and stop events, to figure out their origins, their consequences and their relationships, and to provide a full comprehensive analysis of the natural behavior of C57BL/6J and β2^−/−^ mice. We also investigated the immediate consequences of an acute restraint stress (45 min) applied to the isolated mouse of the dyad on social interactions in C57BL/6J mice and in β2^−/−^ mice, to assess the contribution of β2^−/−^ neuronal nicotinic receptors in this process. We focused on adult animals since β2^−/−^ mice social defects were previously identified in adults (Granon et al., 2003; de Chaumont et al., 2012). As acute stress in adult humans was reported to enhance habit behavior to the detriment of flexible behavior (Starcke and Brand, 2012), we expected here to find reduced social flexibility and more stereotyped actions in both genotypes.

## Materials and methods

### Animals

Male C57BL/6J mice and β2^−/−^ knockout mice purchased from Charles Rivers Laboratories (L'Arbresle Cedex, France) were used in the present study. β2^−/−^ mice were generated from a 129/Sv Embryonic Stem cell line as previously described (Picciotto et al., 1995) and backcrossed onto the C57BL/6J strain for 20 generations. As they were shown to be at more than 99.99% C57BL/6J by a genomic analysis using 400 markers, C57BL/6J mice were used as control of β2^−/−^ animals.

All experimental procedures were carried out in accordance with the European Communities Council Directive of 24 November 1986 (86/609/EEC), EU Directive 2010/63/EU, Decree N 2013-118 of February 1st, 2013, and the French National Committee (87/848). Experiments were conducted in order to reduce the number of mice used and their level of discomfort. C57BL/6J and β2^−/−^ mice, 10–11 weeks old, were group-housed (four mice/cage; size of the cages L: 26.5 cm, l: 16.5 cm, H: 13.5 cm) at their arrival to acclimatize to the animal facilities (food and water *ad libitum*, room temperature 20–22°C) under a 12/12 h light/dark cycle (light on at 7.30 am).

### Behavioral apparatus for social interaction test

The social interaction task was performed in a transparent Plexiglass box (L: 50 cm × l: 25 cm × H: 31 cm) located in an experimental room with light level set at 100–110 Lux by indirect white bulbs. For each experiment, the floor of the cage was covered with clean sawdust. The box was placed under a camera connected to a computer located outside of the experimental room.

### Behavioral protocols and measures

Social behaviors were studied in dyads of male mice composed by an Isolated Host mouse (IH) and a Social Visitor mouse (SV) brought together for the first time. IH mice were individually housed 4 weeks before the social interaction task, while SV mice remained group-housed. IH mice (either C57BL/6J or β2^−/−^ mice) were submitted or not to an acute stress. SV mice were always not stressed C57BL/6J male mice. The choice of 4 weeks social isolation was made after pilot experiments. All behavioral experiments were performed from 9.00 a.m. to 2.00 p.m. (i.e., in the lit phase of the dark/light cycle). Each isolated animal randomly assigned to the stressed groups was placed for 45 min in a Falcon® tube. After 5 min in its home cage, IH mouse was placed in the experimental box for 30 min exploration. Then, a SV mouse was gently introduced in the box, in the corner opposite to the IH mouse. The delay from the beginning of the stress to the end of the social task (i.e., 84 min) was compatible with expression of the c-fos protein in the PFC (Weinberg et al., 2007; Avale et al., 2011), an area previously shown to be critically involved in social interaction (Avale et al., 2011). Control mice were treated similarly, except that they were not submitted to stress (Supplementary Figure 1). In this study, 19 isolated C57BL/6J mice were used (11 were not stressed, i.e., C57BL/6J mice, 8 were subjected to the acute stress, i.e., C57BL/6J Stress mice) and 16 isolated β2^−/−^ mice (9 were not stressed, i.e., β2^−/−^ mice and 7 were submitted to an acute stress, i.e., β2^−/−^ Stress mice). In addition, 35 group-housed C57BL/6J animals served as SV mice.

Social interactions between SV and IH mice were videotaped and analyzed using Mice Profiler software (de Chaumont et al., 2012). Affiliative behaviors studied are explained in Supplementary Table 1. We distinguished contact events, relative positions between mice, dynamic complex events initiated by SV or IH mice, and stop events. Dynamic events were dissociated in first order events (escapes, approaches or follows), second order events (first-order event preceded or followed by a contact) and third order events (2 successions of 2 second-order events). The number and duration of behavioral events of each mouse of the dyad were quantified during the 4 min of the social interaction task.

Chronograms and density graphs were built to illustrate the evolution of the quantitative data in the course of mice dyad interactions. They show behavioral variation of a specific event within an animal and between animals over time. As such, they allow to perceive the stability or instability of given behaviors. For each mouse or each dyad, chronograms depict the frame-by-frame occurrence and duration of a particular event, and thus provide an immediate visualization of its frequency. Density graphs (built from chronograms) represent the temporal evolution of the density of a given event, i.e., the sum of the time in which an event is happening vs. the total time of the bin (grouped here in bins of 30 s). As such, they represent the same information as chronograms but averaged for all mice of a specific group. We also built transitional graphs to illustrate the probability of incidence of one event or posture on the next or the previous one in a sequence.

Finally, we scored dominance and aggressiveness by off line manual analysis. Dominance index was defined as the number of “paw control” (i.e., the number of times IH mouse placed its forepaw on the head or on the back of the SV mouse). Aggressiveness was indexed by the cumulated number of tail rattling and attacks over the 4 min of the experiment. Latencies to first attack and first tail rattling were also scored.

### Plasma corticosterone assays

Biochemical measures of plasma corticosterone were carried out in a pseudo random order by an experimenter blind to mice genotype and stress conditions. Experiments were performed in the same time scale than behavioral ones (10 a.m.–1.30 p.m., i.e., in the lit phase of the dark/light cycle), animals being treated similarly than for behavioral experiments (see Supplementary Figure 1). Trunk blood was collected after mice decapitation. Sacrifices were done 60 min after transfer of mice from animal facility, i.e., after habituation to the experimental room (condition A, *n* = 4 for both genotypes), just after the restraint stress (condition B, *n* = 6 for each genotype) and at the end of the exploration period, i.e., at the time at which social interaction should take place (condition C, *n* = 6 not stressed and 7 stressed C57BL/6J mice, *n* = 7 not stressed and 9 stressed β2^−/−^ mice). Knowing that baseline levels were already measured 60 min after the exit of the animal facility (condition A), waiting 45 min more was extremely unlikely to increase plasma corticosterone levels by itself, so we didn't measure it in additional groups of not stressed animals (condition B). After centrifugation (5000 g 10 min at 4°C), the supernatant was stored at −20°C. Corticosterone levels were quantified using an enzyme immunoassay commercial kit (DetectX®, Arbor Assays). Samples were included into the linear part of the standard curve. Limit of detection was determined as 0.17 μg/dl.

### Statistical analysis

Normal distribution (assessed by Shapiro-Wilkonson test) and equality of variance (evaluated by equal variance test) were first tested using Sigmaplot 12.0. When both were statistically significant, main factor effects and interactions were tested with Two-Way ANOVAs followed by Fisher LSD Method tests when appropriate. When normality and/or equal variance were not statistically significant, Kruskal-Wallis One-Way ANOVA then Mann-Whitney *U*-tests were performed when appropriate. Results were reported as means ± SEM. *P* values ≤ 0.05 were considered statistically significant. Correlations between behavioral events were performed using Spearman test and a Bonferroni correction was applied for multiple comparisons. Statistically threshold was thus set at *p* ≤ 0.0022.

## Results

### Behavioral results

#### Contacts events during social interaction (Figure 1, Table 1)

For similar number, β2^−/−^ mice spent significantly more time in close contact a1 with their partner than C57BL/6J mice [genotype effect: *F*_(1, 31)_ = 5.81, *p* = 0.022] with a stress^*^genotype interaction [*F*_(1, 34)_ = 10.43, *p* = 0.003]. Acute stress only decreased a1 duration in β2^−/−^ mice [stress effect: *F*_(1, 31)_ = 12.77, *p* = 0.001; Fisher LSD Method: *p* < 0.001].

**Figure 1:**
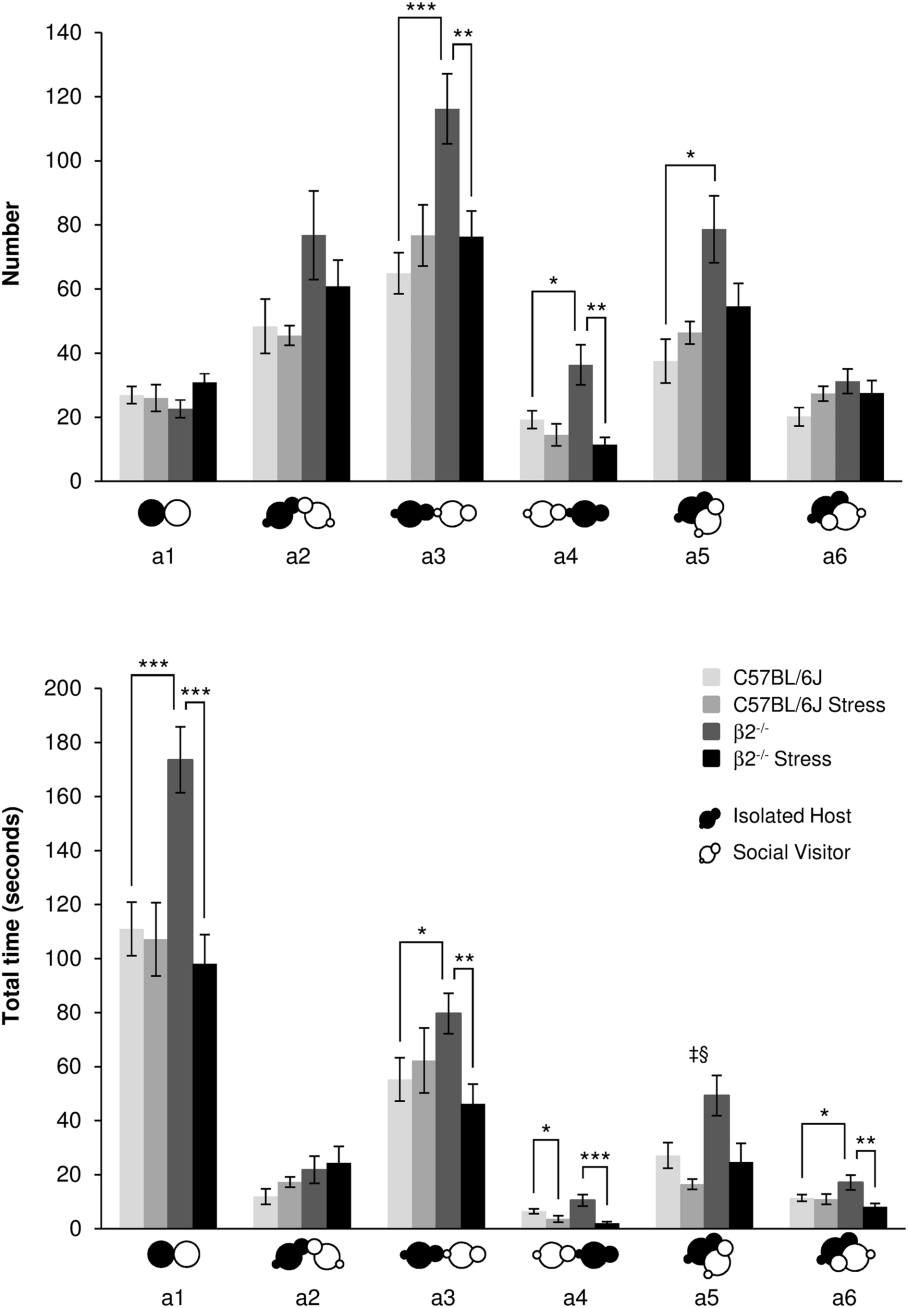
**Contact events during social interaction**. The number (**Top**) and the total time (**Bottom**) of the different types of contact (a1 to a6 as referred in Table 1 and Supplementary Table 1) between IH and SV mice were quantified during the 4 min of the social interaction task. IH mice were submitted or not to an acute stress (C57BL/6J: *n* = 11; β2^−/−^: *n* = 9; C57BL/6J Stress: *n* = 8; β2^−/−^ Stress: *n* = 7). Data are expressed as means ± SEM. ^*^p ≤ 0.05, ^**^p ≤ 0.01, ^***^p ≤ 0.001, ^‡^ Genotype effect: *p* ≤ 0.009, ^§^Stress effect *p* = 0.003.

**Table 1:**
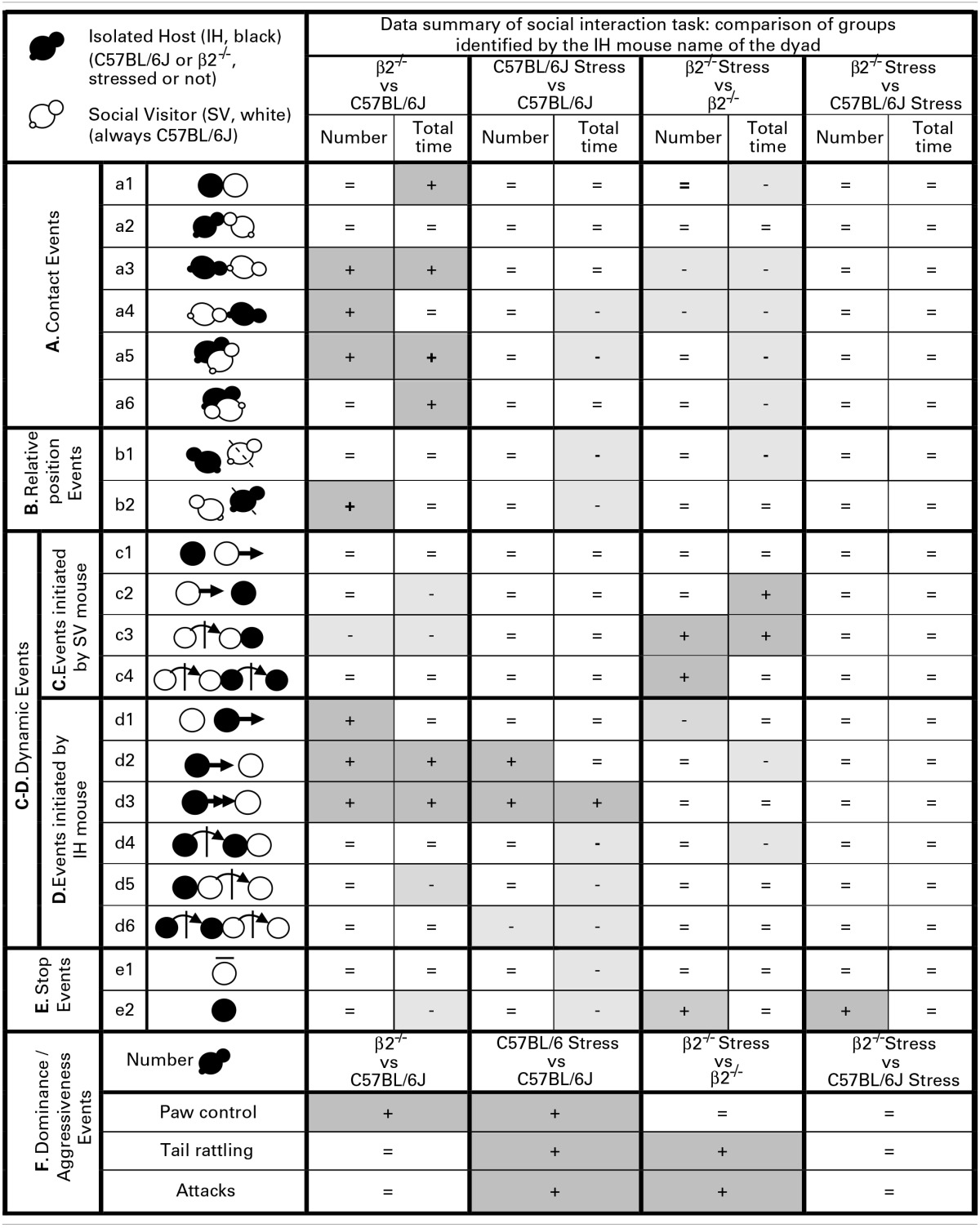
**Data summary of social interaction task**.

*All results were obtained during the social task between a social visitor mouse (SV, always a group-housed C57BL/6J mouse, white symbol) and an isolated host mouse (IH, black symbol). IH mice were either C57BL/6J or β2^−/−^ mice, previously stressed or not. For simplicity reasons, the different tested groups were identified by the IH name of the dyad. Comparisons between the different groups were performed on numbers and times of each behavioral event for the 4 min of the social interaction task. Data are expressed as means ± SEM. Different statistical comparisons were performed: β2^−/−^ mice were compared to C57BL/6J mice, C57BL/6J stressed mice to C57BL/6J mice, β2^−/−^ stressed mice to β2^−/−^ mice and β2^−/−^ stressed mice to C57BL/6J stressed mice. Statistical significant differences between groups were represented by the following signs: +, increase, dark gray overlay; −, decrease, light gray overlay; =, no difference. In the left column, categories, sub - categories and events of the social repertoire (**A–E**, see Supplementary Table 1 for significance of the symbols) as well as IH dominance and aggressiveness **(F)** were presented. Dominance was manually measured by scoring IH's paw control of SV mouse, and aggressiveness by manually scoring IH's tail rattling and attacks. C57BL/6J: *n* = *11*; β2^−/−^: *n* = *9*; C57BL/6J Stress: *n* = *8*; β2^−/−^ Stress, *n* = *7**.

No statistical changes in number and duration for oral-oral contacts (a2 event) were detected (*H* = 5.83, *df* = 3, *p* = 0.12, NS; *H* = 5.49, *df* = 3, *p* = 0.14, NS; respectively).

In regards IH oral-genital contacts with SV mouse (a3 event), β2^−/−^ mice made significantly more such contacts than C57BL/6J mice [*F*_(1, 31)_ = 9.13, *p* = 0.005] with a stress^*^genotype interactions [*F*_(1, 34)_ = 9.47, *p* = 0.004] and no stress effect [*F*_(1, 34)_ = 2.79, *p* = 0.105, NS]. Further Fisher LSD test revealed significant decreased number of a3 event in β2^−/−^ mice after stress (*p* < 0.003). A stress^*^genotype interactions was also found for a3 duration [*F*_(1, 34)_ = 5.66, *p* = 0.024]: a3 event lasted longer in β2^−/−^ mice compared to C57BL/6J mice (Fisher LSD Method *p* < 0.036) and decreased in β2^−/−^ mice after stress (Fisher LSD Method *p* < 0.012).

SV mouse made more oral-genital contacts a4 with β2^−/−^ mice than with C57BL/6J mice (*H* = 13.45, *df* = 3, *p* = 0.004; Mann-Whitney, *p* = 0.024). Acute stress had no effect on this event in C57BL/6J mice but decreased it in β2^−/−^ animals (*H* = 13.45, *df* = 3, *p* = 0.004; Mann-Whitney, *p* = 0.003). Also, acute stress decreased a4 duration both in C57BL/6J (*H* = 17.12, *df* = 3, *p* < 0.001; Mann-Whitney, *p* = 0.043) and β2^−/−^ mice (*H* = 17.12, *df* = 3, *p* < 0.001; Mann-Whitney, *p* = 0.001).

Concerning side-by-side contacts, a5 event increased in number (*H* = 10.80, *df* = 3, *p* = 0.013; Mann-Whitney, *p* = 0.015) in β2^−/−^ mice compared to C57BL/6J animals. No other statistical changes were detected. Duration of this event also increased in β2^−/−^ mice [*F*_(1, 31)_ = 7.85, *p* = 0.009] with no stress^*^genotype interaction [*F*_(1, 34)_ = 1.68, *p* = 0.21, NS] but a stress effect in both genotypes [*F*_(1, 31)_ = 10.61, *p* = 0.003]. Finally, no statistical changes were detected for a6 number. Analysis of a6 duration showed no genotype effect [*F*_(1, 31)_ = 0.67, *p* = 0.42, NS] but a stress^*^genotype interaction [*F*_(1, 34)_ = 5.53, *p* = 0.025]. Fisher LSD Method revealed an increase of a6 duration in β2^−/−^ mice compared to C57BL/6J mice (*p* = 0.022). A stress effect was also detected [*F*_(1, 31)_ = 6.76, *p* = 0.014]: a6 duration decreased after stress in β2^−/−^ mice (Fisher LSD tests: *p* = 0.002).

Overall, events that were increased in β2^−/−^ mice as compared to C57BL/6J were reduced in β2^−/−^ animals after stress and reached C57BL/6J values.

All contact events occurred during the course of the task for all groups of mice (Supplementary Figure 2) but, as a5, they may be less observed at the beginning of the experiment than toward the end. Contact events (a3–a6) decreased from 2 to 2.30 min in C57BL/6J mice, but increased or remained stable in β2^−/−^ mice suggesting that C57BL/6J mice exhibited a progressive decrease of interest for social contacts, and β2^−/−^ mice, a steady one. Also, β2^−/−^ mice, which are affected by the stress, showed a steady interest to contact their partner.

Thus, most contact subtypes were more numerous and lasted longer in β2^−/−^ mice than in C57BL/6J mice. Overall, exposure to restraint stress did not alter contact behaviors of C57BL/6J mice but drastically reduced them in β2^−/−^ mice. They acquired a C57BL/6J social like behavior despite different evolution with time.

#### Relative position events during social interaction (Figure 2, Table 1)

The event b1 (IH mice behind SV mice) occurred similarly in C57BL/6J and β2^−/−^ mice [no genotype effect: *F*_(1, 31)_ = 3.85, *p* = 0.06, NS] for similar time [no genotype effect: *F*_(1, 31)_ = 0.69, *p* = 0.41, NS]. No stress^*^genotype interactions were detected neither for number [*F*_(1, 34)_ = 1.32, *p* = 0.26, NS] or duration [*F*_(1, 31)_ = 0.013, *p* = 0.91, NS] for this relative position event. Stress reduced the total time of b1 event in both genotypes [stress effect: *F*_(1, 31)_ = 4.88, *p* = 0.035].

**Figure 2:**
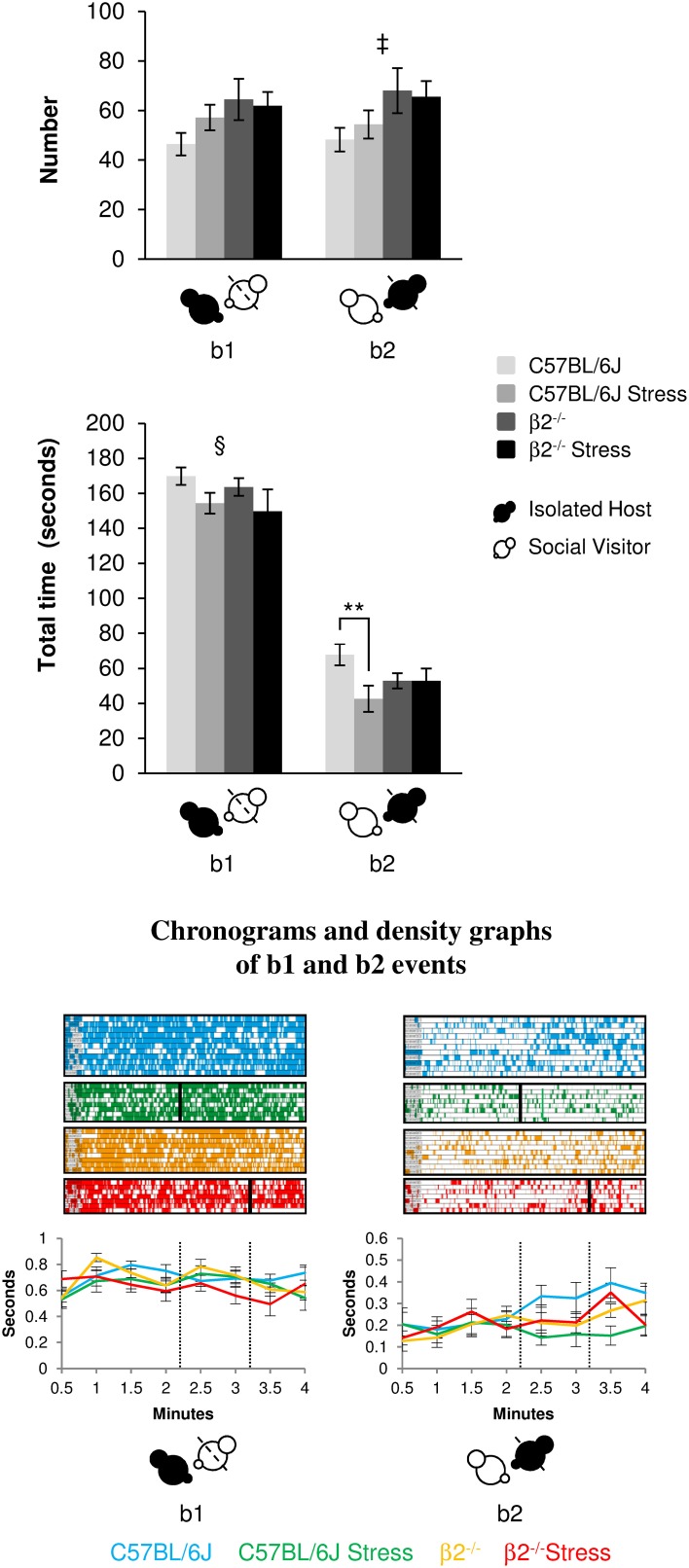
**Relative position events during social interaction**. Histograms (**Top**) showed the number and the total time of the relative position events (b1 and b2 as referred in Table 1 and Supplementary Table 1) between IH and SV, quantified during the 4 min of the social interaction task. IH mice were submitted or not to an acute stress. Data are expressed as means ± SEM. ^**^p ≤ 0.01, ^‡^ Genotype effect: *p* = 0.021, ^§^ Stress effect *p* = 0.035. Chronograms and density graphs (**Bottom**) illustrated the temporal evolution of the aforementioned b1 and b2 events throughout the 4 min of the experiment. In the chronograms, each line represents one dyad of IH - SV mice in a specific group. The length of each point is proportional to the duration of events. In the graphs, each line represented the mean of the temporal evolution of a given event for each group of mice. Vertical lines on chronograms and density graphs indicated the average latency to first attack in stressed C57BL/6J and stressed β2^−/−^ mice (full and dotted lines). C57BL/6J: blue, *n* = 11; C57BL/6J Stress: green, *n* = 8; β2^−/−^: yellow, *n* = 9; β2^−/−^ Stress: red, *n* = 7.

The events b2 (SV mice behind IH mice) occurred more frequently in β2^−/−^ mice as compared to C57BL/6J mice [genotype effect: *F*_(1, 31)_ = 5.94, *p* = 0.021] for similar time [no genotype effect: *F*_(1, 31)_ = 0.15, *p* = 0.70, NS]. Stress^*^genotype interaction [*F*_(1, 34)_ = 4.44, *p* = 0.043] and stress effect [*F*_(1, 31)_ = 4.44, *p* = 0.043] were only detected for b2 duration. Fisher LSD tests showed that acute stress reduced the total time of this b2 event in C57BL/6J (*p* = 0.004) but not in β2^−/−^ mice (*p* = 0.99, NS).

In all groups, b1 event was similarly stable all along the experiment. In contrast, b2 event gradually increased over time in both C57BL/6J and β2^−/−^ mice and remained steadily low in stressed C57BL/6J mice. This suggested that C57BL/6J and β2^−/−^ mice, but not stressed C57BL/6J mice, became used to have their partner behind them.

Thus, all groups of IH mice similarly and continuously preferred to be behind SV mice overtime. Acute stress reduced the total time that mice spent in both positions only in C57BL/6J mice and seemed to prevent stressed C57BL/6J mice to accustom to have their partner behind them.

#### Dynamic events during social interaction

To target specific dynamic behaviors, we dissociated those initiated by SV mice (Table 1C; Supplementary Table 1C) from those initiated by IH mice (Table 1D; Supplementary Table 1D). We dissociated first order events (escapes, approaches, or follows) from second order events (first-order event preceded or followed by a contact) and third order events (2 successions of 2 second-order events).

**Figure 3:**
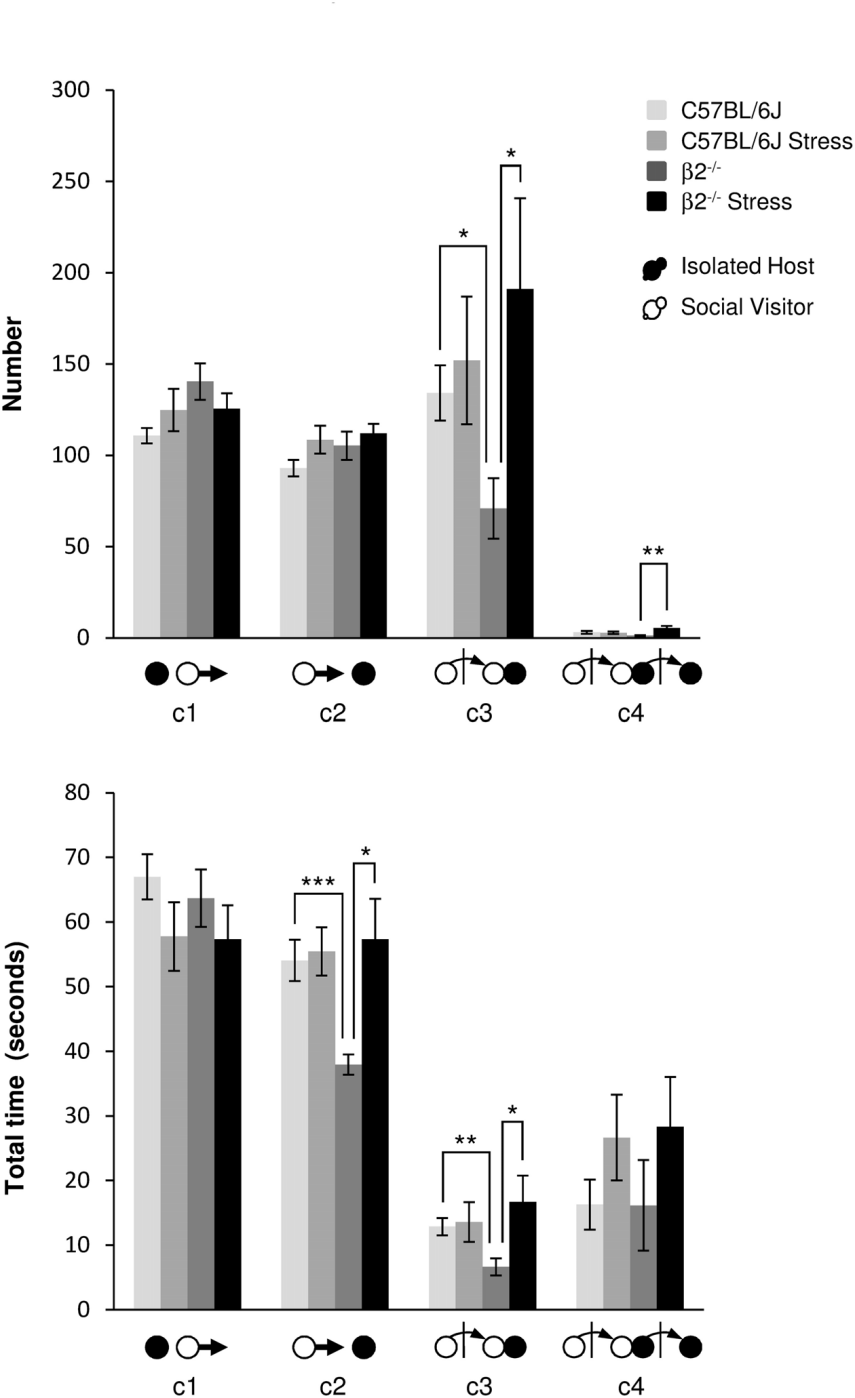
**Dynamic events during social interaction: events initiated by the Social Visitor mouse**. The number (**Top**) and the total time (**Bottom**) of the dynamic events (c1 to c4 as referred in Table 1 and Supplementary Table 1) were quantified during the 4 min of the social interaction task. IH mice were submitted or not to an acute stress (C57BL/6J: *n* = 11; β2^−/−^: *n* = 9; C57BL/6J Stress: *n* = 8; β2^−/−^ Stress: *n* = 7). Data are expressed as means ± SEM. ^*^p ≤ 0.05, ^**^p ≤ 0.01, ^***^p ≤ 0.001.

***Events initiated by the visitor mouse (Figure 3, Table 1)***. For SV escapes number or duration (c1 event), there was no effect of genotype [*F*_(1, 31)_ = 3.57, *p* = 0.07, NS; *F*_(1, 31)_ = 0.19, *p* = 0.665, NS], no stress^*^genotype interaction [*F*_(1, 34)_ = 3.19, *p* = 0.084, NS; *F*_(1, 34)_ = 0.11, *p* = 0.74, NS], nor effect of stress [*F*_(1, 31)_ = 0.002, *p* = 0.964, NS; *F*_(1, 31)_ = 3.30, *p* = 0.079, NS], respectively.

No statistical changes were detected for number of c2 event (IH mouse approached by SV mouse). SV mice approached more quickly a β2^−/−^ than a C57BL/6J mouse (*H* = 14.56, *df* = 3, *p* = 0.002; Mann-Whitney, *p* < 0.001). This event lasted significantly longer in β2^−/−^ mice after stress (*H* = 14.56, *df* = 3, *p* = 0.002; Mann-Whitney, *p* = 0.026).

SV approaches terminated by a social contact (c3 event) were significantly less numerous (*H* = 8.14, *df* = 3, *p* = 0.043; Mann-Whitney, *p* = 0.015) and shorter (*H* = 8.50, *df* = 3, *p* = 0.037; Mann-Whitney, *p* = 0.006) in presence of a β2^−/−^ IH mouse than a C57BL/6J IH mouse. Acute stress significantly increased such SV action (*H* = 8.14, *df* = 3, *p* = 0.043; Mann-Whitney, *p* = 0.03) which lasted significantly longer (*H* = 8.50, *df* = 3, *p* = 0.037; Mann-Whitney, *p* = 0.034) only in presence of a β2^−/−^ mice.

Finally, as regards c4 event, the only statistical change concerned its number that was significantly increased after stress in β2^−/−^ mice compared to not stressed β2^−/−^ mice (*H* = 11.15, *df* = 3, *p* = 0.011; Mann-Whitney, *p* = 0.004).

As a matter of fact, SV actions that were significantly reduced in presence of a β2^−/−^ mice compared to C57BL/6J mice (c3, c4), increased after stress to reach C57BL/6J mice values.

Approaches of SV mice (c2, Supplementary Figure 3) initially similarly highly represented in all groups, decreased quickly and drastically in not stressed animals, and more moderately but linearly in stressed ones to reach similar values at the end of the task. This suggested that SV mice expressed a strong interest for the different IH mice only at the beginning of the experiment and that their interest for their conspecific decreased overtime, whatever is the genotype and the emotional status of their social partner. The rare c4 events, (Supplementary Figure 3), very variable between and within dyads over time, may occur for long periods of time. Notably they may not be exhibited by all dyads, as illustrated by chronograms, and were particularly sporadic when SV faced a β2^−/−^ mice.

Here, we showed that SV approaches, shorter in presence of a β2^−/−^ mouse, were restored after stress. This revealed that the social approaches of SV mice were influenced by the β2^−/−^ genotype and their emotional status.

**Figure 4:**
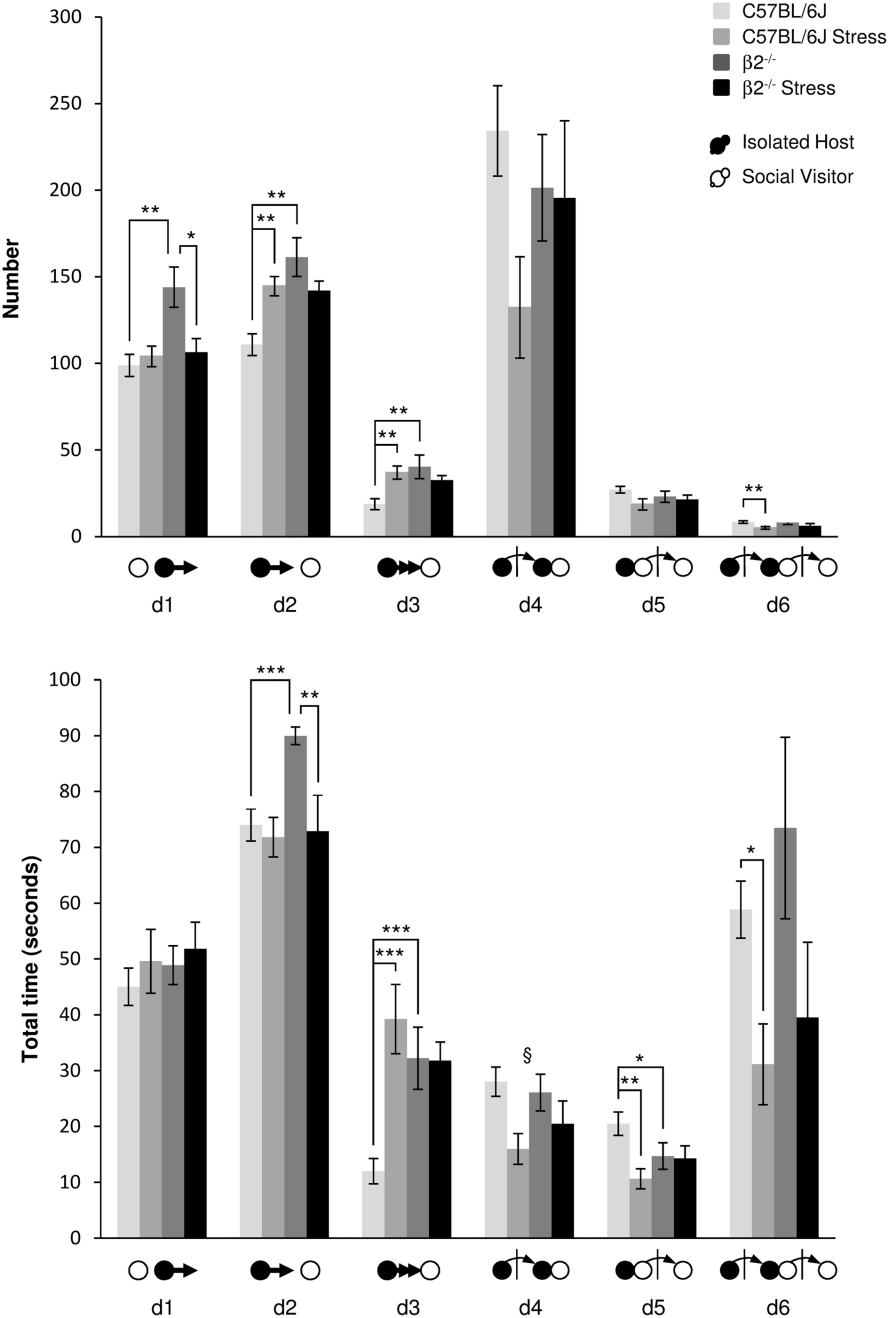
**Dynamic events during social interaction: events initiated by the Isolated Host mouse**. The number (**Top**) and the total time (**Bottom**) of the dynamic events (d1 to d6 as referred in Table 1 and Supplementary Table 1) were quantified during the 4 min of the social interaction task. IH mice were submitted or not to an acute stress (C57BL/6J: *n* = 11; β2^−/−^ : *n* = 9; C57BL/6J Stress: *n* = 8; β2^−/−^ Stress: *n* = 7). Data are expressed as means ± SEM. ^*^p ≤ 0.05, ^**^p ≤ 0.01, ^***^p ≤ 0.001, ^§^Stress effect *p* = 0.006.

***Events initiated by the Isolated Host mouse (Figure 4, Table 1)***. When IH mice were β2^−/−^ animals, the number of first order events significantly increased, e.g., escapes (d1: *H* = 9.99, *df* = 3, *p* = 0.019; Mann-Whitney, *p* = 0.006), approaches (d2: *H* = 16.49, *df* = 3, *p* < 0.001; Mann-Whitney, *p* = 0.003) and follow behaviors (d3: *H* = 12.65, *df* = 3, *p* = 0.005; Mann-Whitney, *p* = 0.009) as compared to C57BL/6J mice. Stress^*^genotype interactions were detected for duration of both d2 and d3 events [*F*_(1, 34)_ = 4.72, *p* = 0.038 and *F*_(1, 34)_ = 10.57, *p* = 0.003, respectively]. Without stress, β2^−/−^ mice spent more time than C57BL/6J mice to approach and follow SV mice (Fisher LSD tests: *ps* = 0.001). In C57BL/6J mice, stress produced an increased number of first order events as approach (d2: *H* = 16.49, *df* = 3, *p* < 0.001; Mann-Whitney, *p* = 0.002) and follow behaviors (d3: *H* = 12.65, *df* = 3, *p* = 0.005; Mann-Whitney, *p* = 0.004). Follows behaviors also lasted longer after stress (d3: stress effect *F*_(1, 31)_ = 9.96, *p* = 0.004, Fisher LSD test *p* < 0.001. In β2^−/−^ mice, almost no significant changes in both number and total time of these first order events were observed after stress, with the exception of decreased in d1 number (*H* = 9.99, *df* = 3, *p* = 0.019; Mann-Whitney, *p* = 0.026), and decreased duration of d2 [stress effect *F*_(1, 31)_ = 7.88, *p* = 0.009, Fisher LSD Method, *p* = 0.002].

Concerning the second order events (d4, d5), no genotype effect [*F*_(1, 31)_ = 0.25, *p* = 0.62, NS], no stress^*^genotype interaction [*F*_(1, 34)_ = 2.52, *p* = 0.12, NS], nor stress effect [*F*_(1, 31)_ = 3.16, *p* = 0.085, NS] were found for d4 number. No stress^*^genotype interaction [*F*_(1, 34)_ = 1.15, *p* = 0.29, NS] but a global stress effect [*F*_(1, 31)_ = 8.73, *p* = 0.006] for the total time of d4 event were detected. Also, for similar number, SV mice spent less time to escape a β2^−/−^ mouse than a C57BL/6J mouse after contact (d5: *H* = 10.58, *df* = 3, *p* = 0.014; Mann-Whitney, *p* = 0.04). Exposing C57BL/6J mice to stress significantly reduced the total time of this d5 event (*H* = 10.58, *df* = 3, *p* = 0.014; Mann-Whitney, *p* = 0.003) while stress had no effect in β2^−/−^ mice.

Finally, the third order events d6 was only affected in C57BL/6J mice after stress. In these mice, stress produced a decreased of d6 number (*H* = 9.35, *df* = 3, *p* = 0.025; Mann-Whitney, *p* = 0.005) and reduced the total time of this events (*H* = 8.82, *df* = 3, *p* = 0.032; Mann-Whitney, *p* = 0.019). No changes in both number and total time of this third order dynamic event were observed in β2^−/−^ mice after stress.

For all groups, d2 events (Supplementary Figure 3) increased in the first minute of the task, earlier and more in not stressed mice of both genotypes than in stressed ones. In contrast, d6 events (Supplementary Figure 3), highly variable between individuals, were more robustly observed in not stressed mice than in stressed animals. With stress, this complex event drastically decreased and may disappear overtime. The near-loss of complex d6 event after stress in C57BL/6J mice could be explained by the drastic drop of d4 and d5 events. In β2^−/−^ mice, it could be linked to the decrease of the simpler event d2.

The only difference between C57BL/6J and β2^−/−^ mice concerned the first order events (approaches and follow behaviors) that were more numerous and lasted longer in β2^−/−^ animals. In C57BL/6J mice, acute stress led to an increase in first order events and to a decrease in second and third ones indicating a reduction in the complexity of behavioral sequences in this strain. In β2^−/−^ mice, stress did not alter the second and third order events but reduced and therefore normalized first order events such as approaches and escapes (Table 1). Results obtained from chronogram analyses further highlighted that stress in C57BL/6J mice touched complex second order events while it altered more simple first order events in β2^−/−^ mice. Thus, stress altered distinct complex behavioral classes in C57BL/6 and β2^−/−^ mice.

#### Stop events during social interaction (Figure 5, Table 1)

The number of stop events of SV mice (e1) was unaffected by the genotype [*F*_(1, 31)_ = 1.95, *p* = 0.17, NS] or the emotional status [stress effect: *F*_(1, 31)_ = 0.013, *p* = 0.91, NS] of the IH mice. No stress^*^genotype interaction [*F*_(1, 34)_ = 0.071, *p* = 0.79] was found. However, SV animals stopped for shorter time in presence of a stressed vs. a not stressed C57BL/6J mouse (*H* = 13.36, *df* = 3, *p* = 0.004; Mann-Whitney, *p* < 0.001).

**Figure 5:**
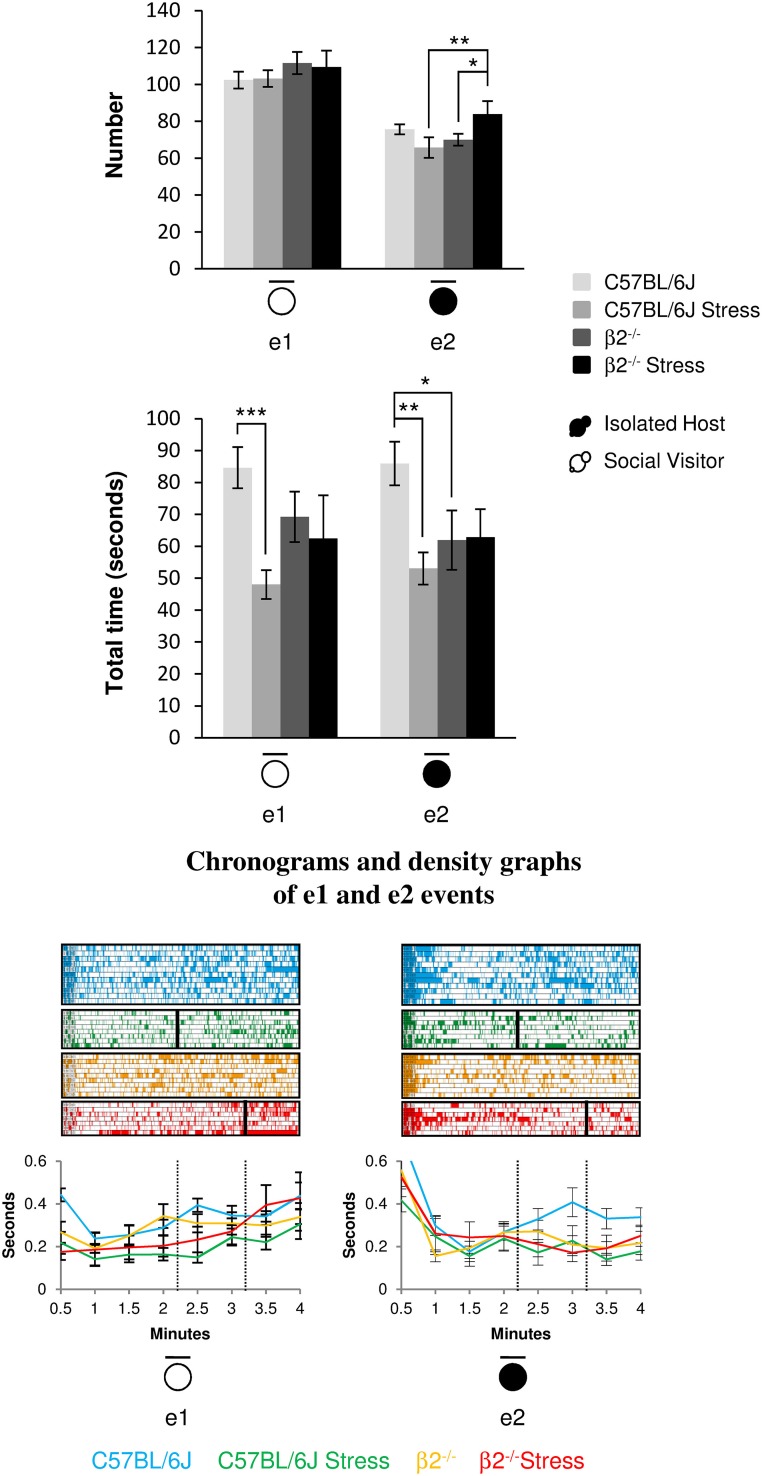
**Stop events during social interaction**. The number (**Top**) and the total time (**Bottom**) of stop events of IH and SV mice in the dyads were quantified during the 4 min of the social interaction task. e1: SV stop events; e2: IH stop events. IH mice were submitted or not to an acute stress. For each group, a corresponding number of SV mice was analyzed. Data are expressed as means ± SEM. ^*^p ≤ 0.05, ^**^p ≤ 0.01, ^***^p ≤ 0.001. Chronograms and density graphs (bottom) illustrated the temporal evolution of the aforementioned e1 and e2 events throughout the 4 min of the experiment. In the chronograms, each line represents stop events of an individual in a specific group. The length of each point is proportional to the duration of events. In the graphs, each line represented the mean of the temporal evolution of a given event for each group of mice. Vertical lines on chronograms and density graphs indicated the average latency to first attack in stressed C57BL/6J and stressed β2^−/−^ mice (full and dotted lines). C57BL/6J: blue, *n* = 11; C57BL/6J Stress: green, *n* = 8; β2^−/−^: yellow, *n* = 9; β2^−/−^ Stress: red, *n* = 7.

For similar duration, no genotype effect was detected in the number of IH stop events (e2) between C57BL/6J and β2^−/−^ mice [*F*_(1, 31)_ = 2.21, *p* = 0.15, NS] but a significant stress^*^genotype interaction was found [*F*_(1, 34)_ = 7.97, *p* = 0.008]. The number of IH stop events increased after stress in β2^−/−^ mice (Fisher LSD Method, *p* = 0.032) but remained unchanged in C57BL/6J animals. In addition, stressed β2^−/−^ made significantly more stop events than stressed C57BL/6J (Fisher LSD Method, *p* = 0.008). Also, for similar numbers, no genotype effect was detected between C57BL/6J and β2^−/−^ mice for e2 stop events duration [*F*_(1, 31)_ = 0.93, *p* = 0.34, NS]. A stress^*^genotype interaction [*F*_(1, 34)_ = 5.36, *p* = 0.027] and a stress effect [*F*_(1, 31)_ = 4.72, *p* = 0.037] were found. Stop events of IH mice were significantly lower in β2^−/−^ mice as compared to C57BL/6J mice (Fisher LSD Method, *p* = 0.018). C57BL/6J mice exposed to an acute stress also spent less time at rest than non-stressed mice (Fisher LSD Method, *p* = 0.002).

Event e1 increased in the first minute of the task in not stressed mice and in the last minute in stressed ones. In contrast, e2 event drastically decreased after the first minute of the task to remain steadily low in all groups except in C57BL/6J mice. This indicates that stop behaviors of the host mice were important feature of an organized and flexible social repertoire in C57BL/6J mice and that they were compromised in animals exhibiting flexibility defects, whether β2^−/−^ mice or stressed animals.

Thus, C57BL/6J mice spent more time at rest than β2^−/−^ mice. Acute stress reduced this time in C57BL/6J mice but had no effect in β2^−/−^ animals. Also, SV mice spent less time at rest in presence of stressed C57BL/6J mice but not when facing a β2^−/−^ mice (stressed or not). Thus, SV mice behavior was only influenced by C57BL/6J behavior.

#### Dominance and aggressiveness during social interaction (Tables 1, 2)

The number of paw control, on one hand, and of tail rattling and attacks, on the other hand, were manually quantified to respectively evaluate the dominance and the aggressiveness of IH mice during social interaction. SV mice never showed tail rattling and never attacked IH mice.

**Table 2:** **Dominance and aggressiveness during social interaction**.

**IH mice**	**C57BL/6J**	**C57BL/6J Stress**	**β2^−/−^**	**β2^−/−^ Stress**
**NUMBER**				
Paw control	5.45 ± 0.98	18.75 ± 3.60^***^	15.56 ± 2.84^†††^	19.29 ± 4.09
Tail rattling	0.00	6.06 ± 3.05^***^	0.00	12.21 ± 6.00^‡^
Attacks	0.00	3.94 ± 1.99^***^	0.00	9.07 ± 4.35^‡‡^
**LATENCY TO FIRST TAIL RATTLING (SECONDS)**				
IH mice	240.00 ± 0.00	149.00 ± 29.72^***^	240.00 ± 0.00	119.57 ± 46.45**^‡‡^**
**LATENCY TO FIRST ATTACK (SECONDS)**				
IH mice	240.00 ± 0.00	169.63 ± 22.14^***^	240.00 ± 0.00	132.71 ± 41.80^‡‡^

The number of times IH mouse controlled SV mouse by placing its forepaw in its head or back (called paw control) was scored manually and defined the IH mouse dominance. Tail rattling, attacks and latencies to their first apparitions were scored to evaluate IH mouse aggressiveness. Values are means ± SEM. Statistical differences between C57BL/6J and C57BL/6J stressed mice were represented by

*** *p* ≤ *0.001*, between C57BL/6J and β2^−/−^ mice by

††† *p* ≤ *0.001* and between β2^−/−^ and β2^−/−^ stressed mice by

‡ *p* ≤ 0.05,

‡‡ *p ≤ 0.01. All comparisons between stressed C57BL/6J and stressed β2^−/−^ mice were not significant. C57BL/6J: n = 11; C57BL/6J Stress: n = 8; β2^−/−^: n = 9; β2^−/−^ Stress, n = 7*.

The number of paw control was significantly higher in β2^−/−^ mice than in C57BL/6J mice (*H* = 18.81, *df* = 3, *p* < 0.001; Mann-Whitney, *p* < 0.001) whereas tail rattling and attacks were absent in both genotypes. After stress exposure, and compared to their not stressed conspecifics, paw control significantly increased in C57BL/6J mice (*H* = 18.81, *df* = 3, *p* < 0.001; Mann-Whitney, *p* = 0.001) but not in β2^−/−^ mice. In contrast, both in C57BL/6J and β2^−/−^ mice, acute stress induced a significant increase in the number of tail rattling (*H* = 17.55, *df* = 3, *p* < 0.001; Mann-Whitney, *p* = 0.001 for C57BL/6J mice, *p* = 0.015 for β2^−/−^ mice) and attacks (*H* = 19.92, *df* = 3, *p* < 0.001; Mann-Whitney, *p* = 0.001 for C57BL/6J mice, *p* = 0.005 for β2^−/−^ mice). The range of tail rattling numbers was comprised between 0 and 25 in stressed C57BL/6J mice and between 0 and 35 in stressed β2^−/−^ mice while the number of attack was comprised between 0 and 15 in stressed C57BL/6J mice and between 0 and 28 in stressed β2^−/−^ mice. Even though quantitative expression of aggression varied individually, a large majority of mice exhibited aggressive behavior after stress whatever their genotypes (Supplementary Table 2). In addition, latency to first attack from stressed C57BL/6J mice was longer (in about 3 min) but not significantly different than that stressed β2^−/−^ animals (in about 2 min).

Thus, restraint stress increased dominance only in C57BL/6J mice, and induced aggressiveness in both mouse genotypes. Aggressiveness was not due to previous mice isolation since it did not exist in not stressed mice. As such, it was imputable to stress and was independent of nicotinic system.

#### Correlations between behavioral events during social interaction (Figure 6)

**Figure 6:**
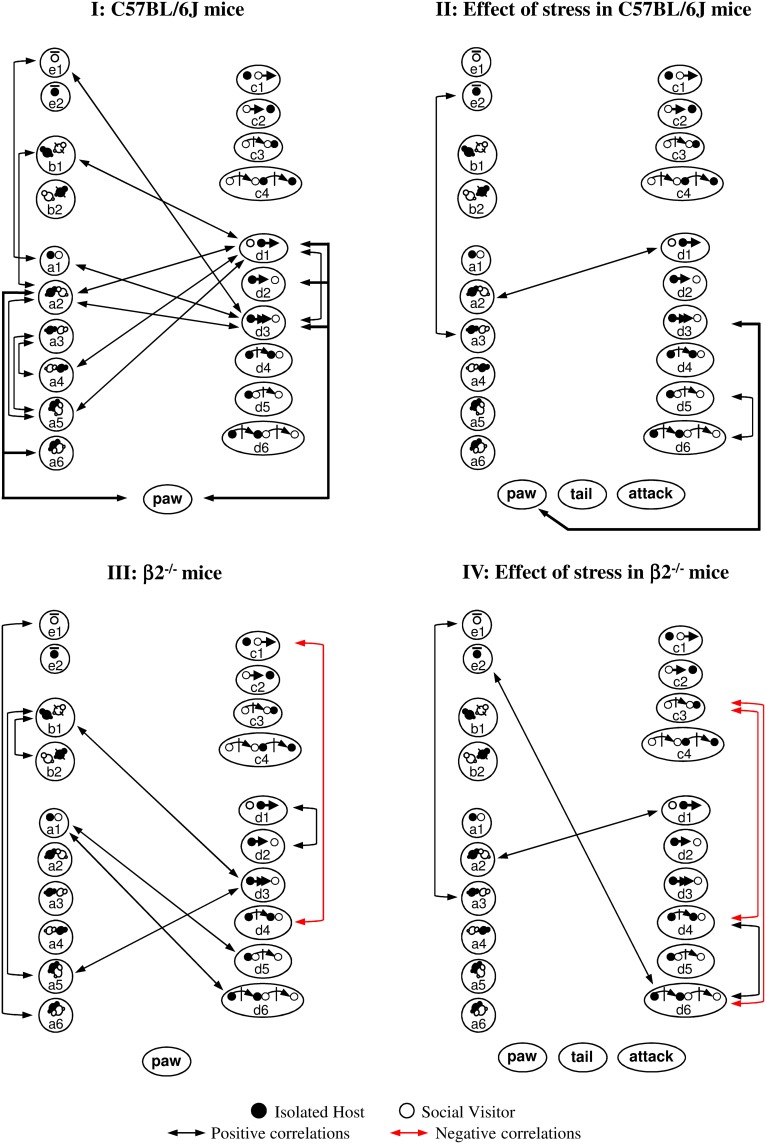
**Correlations between behavioral events during social interaction**. Contact, mice position and stop events were represented on the left, sequential complex events on the right, dominance and aggressiveness at the bottom. Black arrows indicated positive correlations (0.0000002 ≤ *p* ≤ 0.002), red arrows negative ones (*p* = 0.0000002). **(I)** C57BL/6J mice. All correlations were positive. Many contact events were correlated between them and some with d3 or d1 events. Events initiated by C57BL/6J mice were interrelated (d3-e1; d3-d1). Social dominance was correlated to many events (see also Supplementary Figure 4I). **(II)** Effect of stress in C57BL/6J mice. Very few correlations were detected in stressed C57BL/6J mice. Contact events became almost completely disconnected from follow and escape behaviors. Paw control, more exhibited by C57BL/6J stressed animals (Tables 1, 2), became only linked with follow behaviors (d3, see also Supplementary Figure 4I). Although aggression-like behaviors emerged, no correlation with any social or dominance events were detected. **(III)** β2^−/−^ mice. Compared to C57BL/6J mice, contact events were not interrelated anymore. Correlations within other behavioral categories appeared. Follow events became interrelated with a5 and b1 events (see also Supplementary Figure 4II). Even if paw control was significantly more abundant in β2^−/−^ mice than in C57BL/6J mice (Tables 1, 2), this index of dominance was not correlated with any event. **(IV)** Effect of stress in β2^−/−^ mice. All correlations observed in β2^−/−^ mice disappeared after stress while some other positive ones emerged (see also Supplementary Figure 4III) Follow behaviors, not quantitatively modified by stress (Figure 4) became disconnected from all events. All index of dominance and aggression correlated with none behavioral events in the stressed β2^−/−^ mice social repertoire even if tail rattling and aggression drastically increased (Table 2). See Supplementary Table 1 for behavioral symbols. Correlations were taken into account for *p* ≤ 0.0022. C57BL/6J: *n* = 11; β2^−/−^: *n* = 9; C57BL/6J Stress: *n* = 8; β2^−/−^ Stress: *n* = 7.

Statistical correlations we performed to identify putative relationships between the different events of the mice social repertoire within each group of mice. Supplementary Figure 4 illustrated correlations for some key events and Figure 6 showed all significant statistical correlations.

With C57BL/6J mice, SV mice actions did not participate in the generation of contact or complex events (**I**). This data suggested that the host mouse was the principal actor in the dyad as it generated most contacts and was dominant. In addition, it showed that dominance (“paw”) was associated with high sociability (i.e., various types of contact, approach, escape). After stress, most social relationships in C57BL/6J mice were broken while behavioral correlates of dominance diminished (**II**). Social organization in β2^−/−^ mice appeared totally different (in particular for contact events) and reduced compared to C57BL/6J mice with dominance totally disconnected from social behavior except from follow behavior (**III**). After stress, an impoverishment and a marked fragmentation of social behavior in β2^−/−^ mice appeared (**IV**).

Thus, we evidenced drastic differences in the arrangement of social events between C57BL/6J and β2^−/−^ mice. Acute stress deconstructed the links existing between the different events of a given behavioral class and between the various classes of the social repertoire in both genotypes. This occurred even if there is no quantitative difference in the social repertoire and in the emergence of aggressiveness in stressed C57BL/6J and stressed β2^−/−^ mice (Table 1). In addition, although dominance normally exists in both C57BL/6J and β2^−/−^ not stressed animals, its value and consequences were only modified after stress in C57BL/6J mice.

#### Behavioral transitions during social interaction (Figure 7)

Transition graphs were built to investigate how the various events fit together during the social interaction task. They represent the probability of a given event to be preceded or followed by one or several events and provide complementary information from quantitative data. Indeed, d3 event, poorly represented in number or time in all groups (Figure 4), will appear to be crucial to trigger behavioral strings (see below). From the repertoire previously described (Supplementary Table 1), the key behavioral events participating to the dyads actions were contact (a1, a2, a3, and a4), relative position (b1 and b2), follow (d3), stops (e1, e2) and dynamic (d6) events.

**Figure 7:**
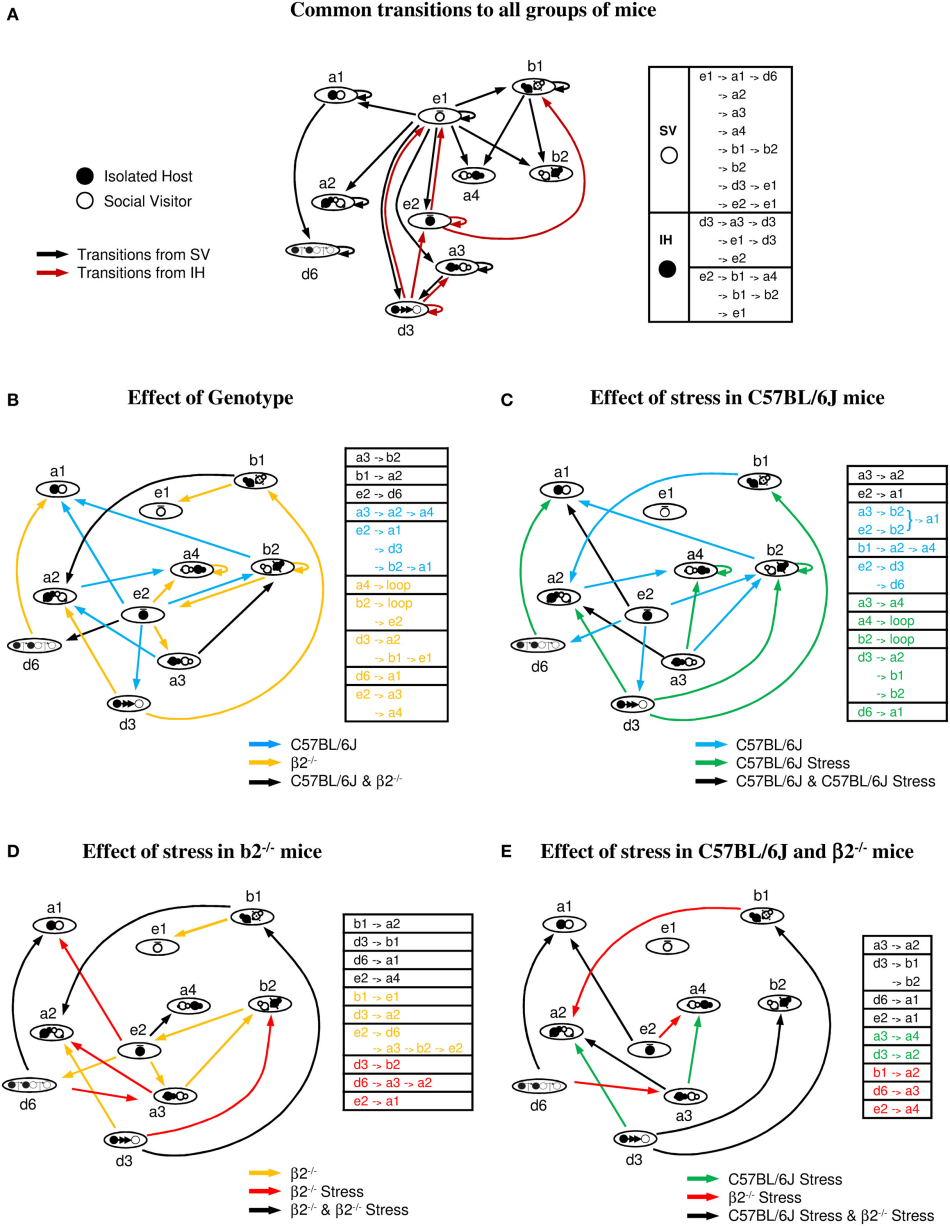
**Behavioral transitions during social interaction. (A)** Common transitions to all groups of mice: major sequences common to stressed or not stressed C57BL/6J and β2^−/−^ mice. Transitions initiated by SV mice are indicated by black arrows, those initiated by IH mice by brown arrows. **(B)** Effect of genotype: common (black arrows) and specific transitions to C57BL/6J (blue arrows) and β2^−/−^ mice (yellow arrows). **(C)** Effect of stress in C57BL/6J mice: common (black arrows) and specific transitions to C57BL/6J (blue arrows) and C57BL/6J stressed mice (green arrows). **(D)** Effect of stress in β2^−/−^ mice: common (black arrows) and specific transitions to β2^−/−^ (yellow arrows) and β2^−/−^ stressed mice (red arrows). **(E)** Effect of stress in C57BL/6J and β2^−/−^ mice: common (black arrows) and specific transitions to C57BL/6J stressed (green arrows) and β2^−/−^ stressed mice (red arrows). Tables next to each figure summarized the common and the specific transitions of each group of mice. See Supplementary Table 1 for significance of the symbols.

***Common transitions to all groups of mice (Figure 7A)***. As shown in Figure 7A, dyads of all groups shared multiple sequences of action. In addition, the majority of social behaviors displayed a repetitive pattern (i.e., high probability to occur at least twice successively) suggesting a strong value for the given behavior.

In all groups, stops of the SV mouse (e1, black arrows) triggered all main contacts, postures, and dynamic events of the dyads. It suggested that stops of the SV mouse triggered fixed behaviors whatever the IH mouse it faced. This occurs even if SV mice spent less time at rest in presence of a stressed than a not stressed C57BL/6J mouse (Figure 5, e1, bottom). In contrast, all IH mice shared 2 behaviors initiating other behavioral sequences: stops (e2) and follow behaviors (d3) (brown arrows). The event e2 had two main consequences, e1 (SV stops) and b1 (SV in front of IH) while d3 event had three major consequences, both IH and SV stops (e1 and e2) and oral-genital contacts (a3).

SV mice were always C57BL/6J group-housed not stressed mice. Therefore, independently of the genotype or the emotional status of the social partner, common transitions between key behavioral events were centered on stops of the SV mice. Moreover, host mice from the different groups, which were always previously isolated and habituated to the environment, shared two key behavioral events: stops and follow behaviors.

In addition to common transitions described above, the different groups of mice share or not some transitions (see below).

***Effect of genotype (comparisons between C57BL/6J and β2^−/−^ mice Figure 7B)***. Some sequences were observed both in C57BL/6J and in β2^−/−^ mice (black arrows) while others were specific either to C57BL/6J (blue arrows) or to β2^−/−^ mice (yellow arrows).

C57BL/6J stop events (e2) triggered close contact (a1), relative position (b2), and follows (d3) events. In contrast, stop events of β2^−/−^ mice (e2), which lasted significantly less than that of C57BL/6J mice (Figure 5), only induced oral-genital contacts (a3 and a4).

Thus, stop events played a crucial role in the initiation of numerous different behaviors in C57BL/6J mice. In contrast, follow behaviors appeared to play a fulcrum role in β2^−/−^ animals.

***Effect of stress in C57BL/6J mice (Figure 7C)***. Both stressed and not stressed C57BL/6J mice shared some sequences indicating that they are independent of the emotional status of the mice (black arrows).

In C57BL/6J mice, long sequences existed, while acute stress induced shorter ones. In not stressed mice, stop events (e2) triggered several sequences (blue arrows) while in stressed mice, e2 event which lasted significantly less than in C57BL/6J mice (Figure 5), induced only one (green arrows). This is in concordance with the aforementioned key role of e2 event in not stressed C57BL/6J mice. This important e2 fulcrum is almost abolished by stress.

Interestingly, some strings only observed after stress in C57BL/6J animals existed in β2^−/−^ mice. Also, d3 event in stressed C57BL/6J mice appeared crucial to initiate behavioral sequences as seen for β2^−/−^ mice. Thus, these results complemented our quantitative data showing some similarities of social behavior between stressed C57BL/6J and not stressed β2^−/−^ mice.

Overall, acute stress shortened behavioral sequences in C57BL/6J mice and dramatically triggered aggressive behaviors (Table 2). Moreover, the social repertoire partially shared by C57BL/6J after stress mice and β2^−/−^ mice supported the idea of a close behavioral organization in the two groups.

***Effect of stress in β2^−/−^ mice (Figure 7D)***. β2^−/−^ and β2^−/−^ stressed mice shared some sequences that are likely to reflect the core of the β2^−/−^ social repertoire (black arrows). These common transitions were more numerous than in stressed and not stressed C57BL/6J mice, reinforcing a more rigid behavior in β2^−/−^ mice than C57BL/6J mice.

Some transitions observed in stressed and not stressed β2^−/−^ mice existed in stressed C57BL/6J mice (Figure 7C) while some transitions appearing after stress in β2^−/−^ mice existed in stressed and not stressed C57BL6 mice. Additional sequences, seen in C57BL6 stressed mice appeared after stress in β2^−/−^ mice while others disappeared. These results were in concordance with our quantitative analysis showing similar data between C57BL6 and β2^−/−^ mice after stress (Table 1). In addition, stress diminished the crucial role of e2 event in β2^−/−^ mice as partially seen in C57BL6 mice for which e2 event triggered no sequences, aside from that shared by all groups (Figure 7A).

Overall, acute stress in β2^−/−^ mice induced behavioral transitions common to the C57BL6 strain or specific to all stressed animals. These data and quantitative results (see Table 1) support the idea that after acute stress, social behavior of β2^−/−^ mice acquired C57BL/6J features.

***Effect of stress in C57BL/6J and in β2^−/−^ mice (Figure 7E)***. All stressed mice shared most transitions (black arrows) while very few transitions differed between stressed C57BL/6J (green arrows) and stressed β2^−/−^ animals (red arrows). Interestingly, acute stress induced in all mice shrinkage of behavioral transitions as evidenced by correlation analyses. After stress, behavioral sequences in both mouse genotypes tend to be similar in coherence with the quantitative results showing no statistical difference in the behavioral repertoire, dominance and aggressiveness between stressed C57BL/6J and stressed β2^−/–/−^ mice (Tables 1, 2).

#### Plasma corticosterone levels (Table 3)

Plasma corticosterone levels were measured as described in Supplementary Figure 1. Statistical analyses showed a significant group effect (*H* = 27.17, *df* = 7, *p* < 0.001). Mann-Whitney tests further revealed no significant genotype effect in condition A (measures performed 60 min after mice transfer from animal facility, *p* = 0.69), in condition B (measures performed immediately after stress, *p* = 0.13), and in condition C (measures performed after exploration) for stressed (*p* = 0.67) and not stressed mice (*p* = 0.29). In contrast, whatever the mice genotype, not stressed animal showed significant increases in plasma corticosterone levels in condition C compared to condition A (*p* = 0.01 in C57BL/6J mice and *p* = 0.006 in β2^−/−^ mice). Also, a significant stress effect was observed when comparison were performed between conditions A and B (*ps* = 0.01 in both C57BL/6J and β2^−/−^ mice) or between conditions A and C (*p* = 0.006 in C57BL/6J mice and *p* = 0.007 in β2^−/−^ mice). Finally, a significant decreased was detected in β2^−/−^ mice at the end of the exploration (comparison conditions C vs. B, *p* = 0.02). Thus, whatever the mice genotype and its emotional status (stressed or not), corticosterone levels measured after exploration (condition C) were higher than those measured at the exit of the animal facility.

**Table 3:** **Plasma corticosterone levels (μg/dl) in different experimental conditions**.

**Mice**	**Condition A**	**Condition B**	**Condition C**
C57BL/6J	1.21 ± 0.48		12.73 ± 0.98^††^
C57BL/6J Stress		13.42 ± 2.42^*^	11.85 ± 1.79^**^
β2^−/−^	1.11 ± 0.79		10.23 ± 1.72^†††^
β2^−/−^ Stress		20.10 ± 2.75^*^	13.55 ± 1.66^**^, ^‡^

Blood samples were collected in three different experimental conditions: condition A, 60 min after mice exit from animal facility (n = 4 for each genotype); condition B, after stress (n = 6 for each genotype), and condition C, after the exploration period (C57BL/6J mice: n = 6 not stressed, n = 7 stressed; β2^−/−^ mice: n = 7 not stressed, n = 9 stressed). Values are means ± SEM. Effects of stress within genotype:

* p = 0.01,

** p ≤ 0.007. Effect of condition within genotype in not stressed (

†† p = 0.01;

††† p = 0.006) and stressed mice (

‡ *p = 0.02)*.

## Discussion

The present study was performed to determine the immediate impact of acute restraint stress on social interactions in adult male mice and to investigate the contribution of neuronal nicotinic receptors (nAChRs) in these effects. C57BL/6J mice were used, because they were known to exhibit high sociability (Sankoorikal et al., 2006) and are largely used by the Neuroscience community. β2^−/−^ mice which constitutively lack nAChRs were used because they expressed less behavioral flexibility in social contexts (Granon et al., 2003; Besson et al., 2008).

### Core social behaviors independent of genotype and stress status

We found that, in all 4 types of dyads, SV stops always occurred at the starting point of contact events, postures, or dynamic event, regardless of the emotional status or genotype of its partner. In addition, for all dyads, IH stops and follow events were key points in the social repertoire.

### Exhaustive analysis: behavioral similarities and differences in C57BL/6J and β2^−/−^ mice

We first confirmed here previous data as β2^−/−^ mice made longer contacts and more numerous follows, spent less time at rest and were more dominant (Granon et al., 2003; Maubourguet et al., 2008; de Chaumont et al., 2012; Coura et al., 2013). We also confirmed that key events, crucial for initiating various behaviors, differed between the two mouse genotypes. We thus explored thoroughly their social consequences in both genotypes. We found that β2^−/−^ mice exhibited a strong and constant interest for contacting their social partner as the experiment progressed while C57BL/6J animals, decreasing their interests for social contact, became accustomed to the social situation over time (Supplementary Figure 2). Furthermore, follows in β2^−/−^ mice were only correlated with oral-oral contact and posture behind the SV mouse while they were correlated with various actions (contacts, escape, dominance) in C57BL/6J mice (Figure 6). This indicated that follows are associated to a restricted social repertoire in β2^−/−^ mice but to a large panel of behavior with no fixed consequences in C57BL/6J. Also, correlation analyses showed no links between different contact subtypes in β2^−/−^ mice while many of them were related in C57BL/6J animals (Figure 6). Stop events allowed mice to scan their environment and to acquire and evaluate information about potential risks (Quartermain et al., 1996; Maubourguet et al., 2008). We showed here that their relative absence in β2^−/−^ mice, consistent with their hyperactivity (Avale et al., 2008), had strong consequences on the social repertoire: in β2^−/−^ mice, stop events only induced oral-genital contacts while in C57BL/6J mice, stop events triggered multiple events, including contacts, relative position events, and follows (Figure 7). All these data reinforced the fact that β2^−/−^ mice exhibited less flexible behavior than C57BL/6J and showed more stereotyped and restricted actions (Granon et al., 2003; Maubourguet et al., 2008; de Chaumont et al., 2012). It also showed that the behavioral repertoire may be possessed by both genotypes but may not have the same “value.”

We revealed a crucial importance of dominance behaviors: in C57BL/6J mice dominance was associated with a large panel of social contact but this was not the case in β2^−/−^ mice, for which dominance seemed an isolated compound (Figure 6), although quantitatively over represented (Table 2). This data matched with our previous findings, which showed that dominance tendencies were strongly modulated by the nicotinic-cholinergic system (Coura et al., 2013). For the first time, we showed that behavioral differences between C57BL/6J and β2^−/−^ mice were not due to different plasma corticosterone levels, either measured in a control condition (60 min after exit from animal facility), or after exploration of the novel environment. Baseline measures were very low, in agreement with others results (Thoeringer et al., 2007). This showed that animal isolation *per se* did not generate a chronic stress. However, as compared to baseline values, novelty exploration increased corticosterone in all not stressed animals (Table 3), as had been reported by others in C57BL/6J animals (Roozendaal, 2002; Chauveau et al., 2010; Beerling et al., 2011; Chaouloff and Groc, 2011), showing that exploration of a novel environment induced activation of physiological parameters normally associated to stress responses (De Boer and Van der Gugten, 1987; Beerling et al., 2011). Besides, we detected that both mouse genotypes similarly and largely preferred to be behind SV mice all along the experiment. In contrast, the opposite posture gradually appeared more often over time, particularly in C57BL/6J mice (Figure 2). These findings can be explained by the fact that SV mice were always C57BL/6J, group-housed, not stressed and never dominant in our experimental condition. Indeed, it was shown that taking place behind a partner is a posture likely to be exhibited by dominant animals (Arakawa et al., 2007). Thus, our results suggested that relative positions events in this task could be used as an index of a mouse's tolerance to another mouse.

In summary, the present study showed that each mouse genotype exhibited a specific complex social behavioral pattern and developed distinct social strategies. It highlighted major behavioral difference between C57BL/6J and β2^−/−^ mice, hence the important role of the cholinergic nicotinic system in the social strategy used by animals to organize their behavior. The differences between C57BL/6J and β2^−/−^ mice in this task may rely on a different trade-off between exploitation and exploration: C57BL/6J mice would favor exploration of various options, including social contact when confronted to a novel environment, and β2^−/−^ mice would favor exploitation of the social reinforcement. Favoring exploitation over exploration has been shown to be accompanied by rigid behaviors (Cohen et al., 2007). This view is consistent with the involvement of the monoaminergic ascendant system on one hand and of the cholinergic system on the other hand in the governance of the balance between exploration and exploitation (Cohen and Aston-Jones, 2005; Yu and Dayan, 2005). As the regulation of this balance has been shown fundamental for adaptive and flexible behaviors (Kehagia et al., 2010), our data supported the need for functional nAchRs in social context, complex, changing and uncertain by nature (for review, Cohen et al., 2007).

### Stress effects in C57BL/6J mice

Although SV mice were not subjected to stress procedure, it indirectly altered their behavior: it shorted their stops and their tolerance when facing a stressed C57BL/6J mouse, indicating that SV was influenced by the emotional status of its partner. This can be due to the increase of follows, dominance and aggressiveness exhibited by stressed mice. This adaptation to their social partner, in turn, deeply modified their entire social repertoire as seen in transition graphs. In another social paradigm (3 chambers), Yang et al. (2012) showed that C57BL/6J mice adjusted their behavior, depending on the genotype of their partner. Also, Varlinskaya et al. (1999) showed that adolescent rats changed their social behaviors depending on the social activity of their play partner, as observed in other species (for review, Blanchard et al., 2003) or in other social tasks (Nyberg et al., 2004; Moy et al., 2008; Fairless et al., 2013). However, this is the first study to report that such a profound, dynamic and rapid adaptation occurred in a dyad of adult mice that had never interacted before. Future studies should investigate how this adaptation might be achieved; it may involve communication, via acoustic (Panksepp and Lahvis, 2011; Chabout et al., 2013), olfactory (Arakawa et al., 2008a,b; Deschenes et al., 2012), visual (Clark et al., 2006), and/or tactile (Clark et al., 2006; Wolfe et al., 2011; Deschenes et al., 2012) cues (Adolphs, 2010; Silverman et al., 2010).

Stress had three main consequences in IH C57BL/6J mice: it impoverished the social repertoire, increased dominance, and fostered aggressive behaviors. The social impoverishment was illustrated by the reduction of complex sequences with concomitant increase in single events such as approaches and follows, the shortening of some contact events and of mutual tolerance (Table 1). Stress also reduced the duration of stop events that were shown to be keys in the decision process for making various social actions. In consequence, their impact on subsequent social behaviors was abolished (Figure 7). Therefore, by shortening the time spent scanning their environment during stops, stressed mice reduced the panel of their possible behavioral choices. Interestingly, such stress effect has also been reported in humans to promote habits (Schwabe and Wolf, 2009) and to reduce the panel of strategies during decision-making tasks (for review, Starcke and Brand, 2012). Also, we showed that follow behaviors increased after stress and appeared crucial for initiating multiple short sequences. Their consequences changed as compared to not stress condition: the relationships between contacts and follows were completely abolished by stress (Figure 6). These features were those observed in β2^−/−^ mice without stress. As consequence, the complexity and the length of behavioral sequences were reduced in stressed C57BL/6J mice, and social behaviors appeared scattered and less fluently organized. Our data thus showed that the meaning of behaviors that are part of the normal social repertoire (e.g., follows) may depend on the emotional status of the animals. It implies that the gross measurement of their existence is not sufficient to characterize a social deficit in mice models.

We reported that single restraint stress increased dominant behaviors by more than three-fold in C57BL/6J mice, and dramatically altered their consequences. Social dominance is a normal behavior (Blanchard et al., 1984, 2003; Chichinadze et al., 2011). Without stress, dominance promotes social contacts whereas after stress, it favored only follows (Figure 6).

We showed that aggressiveness and dominance were dissociated social features that were never correlated, as previously reported (Coura et al., 2013). IH mice were isolated for 4 weeks prior to the experiments. It is questionable whether social isolation can itself promote aggression. To investigate this question, we compared our social task with other tasks studying aggressiveness. For example, in the resident-intruder task, classically used to trigger aggressiveness, social isolation is not the only component of the procedure: aggression is typically realized in the home cage of the isolated mouse and in some cases, males are previously exposed to females to increase territorial behavior, or are confronted with partners of a different size (Miczek and O'Donnell, 1978; Crawley, 2000; Miczek et al., 2001; Arregi et al., 2006; Neumann et al., 2010; Yohe et al., 2012; Marquez et al., 2013). In the present study, the task was never conducted in the home cage of a mouse. Also, the experimental cage was much larger than home cages, and it contained clean bedding; this environment favored novelty exploration and prevented strong territorial behavior as shown previously (Supplementary Figure 1 in Avale et al., 2011). Finally, our animals were never exposed to females. Therefore, our task differed dramatically from other tasks widely used to trigger aggressiveness. Moreover, we used C57BL/6J mice, which exhibit high sociability (Sankoorikal et al., 2006; Yang et al., 2012) and are generally not considered an aggressive strain (Puglisi-Allegra and Cabib, 1985; Roubertoux et al., 1999; Le Roy et al., 2000; Sankoorikal et al., 2006; de Chaumont et al., 2012), even after 8 weeks of social isolation (Puglisi-Allegra and Cabib, 1985). This non-aggressive disposition was confirmed in our study, because no aggression was observed in not stressed dyads. Thus, stress-induced aggressive behavior could not be attributed to the isolation of one of the adult mice in our experimental conditions. Also, in social contexts, aggression can be promoted by various experiences, including shocks (Puglisi-Allegra and Cabib, 1985), post-weaning isolation (Toth et al., 2011, 2012; Tulogdi et al., 2014), post-traumatic stress disorders (Lasko et al., 1994; Pavic et al., 2003; Haller et al., 2005), and early life stressful experiences (Veenema, 2009; Marquez et al., 2013). Prolonged or recurrent stress also contributed to aggressive behaviors (Wood et al., 2003; Yohe et al., 2012; Umukoro et al., 2013). To our knowledge, however, the present study was the first to assess aggressive behavior after delivering a single short stress to adult mice. As expected, immobilization stress increased plasma corticosterone levels. It is noticeable that plasma corticosterone levels remained steadily high when stress was followed by novelty exploration in C57BL/6J mice. Therefore, our stress procedure did not superimpose to a putative isolation-induced stress.

Social experiments reported by others were mostly performed during the light phase of the cycle, although some experiments were conducted during the dark phase. However, it has been reviewed recently that sociability in rodents can be efficiently evaluated in the light phase (Yang et al., 2007, 2008). It is well known that plasma corticosterone has a circadian rhythm, with a peak around 3 p.m., a largest second one around 7 p.m., and a relatively high level during the first 6 h of night (De Boer and Van der Gugten, 1987; Kalsbeek et al., 1996). However, we cannot exclude that our experiments, conducted when plasma corticosterone levels were the lowest, could have led to different behavioral results if conducted in the dark phase of the cycle. In our experimental conditions, aggressiveness cannot be due to changes in plasma corticosterone levels induced by novelty exploration nor to the stress itself. Since social behaviors are supported by the HPA axis (for review, Owings and Coss, 2007), it may be involved, at least partially, in the stress responses observed here. But how the adaptive adjustment of behavioral system develops and evolves remains to be determined.

In conclusion, stressed C57BL/6J mice managed social contact with a novel social partner by reorganizing their behavior; moreover, they reduced or eliminated complex sequential behaviors. Interestingly, the dominance level in stressed C57BL/6J mice increased to the levels observed in β2^−/−^ mice. This finding suggested that stress may alleviate behavioral inhibition mediated by the cholinergic system (Picciotto et al., 2012) or by the prefrontal monoaminergic systems (Arnsten, 2009; Ginsberg et al., 2011), because β2^−/−^ mice exhibited constitutive major increases in the monoaminergic and cholinergic tones in the prefrontal cortex (PFC) (Coura et al., 2013). Previous studies showed that aggressiveness also resulted from lesions in the NA system (Cambon et al., 2010) or NA PFC depletion (Coura et al., 2013), and was associated with brain cholinergic and NA systems (Maxson, 2009; Ginsberg et al., 2011). Thus, the effects of stress in C57BL/6J mice may have been due to an acute increase in acetylcholine and/or monoamine PFC tone (Das et al., 2000; Del Arco and Mora, 2009; for review, Picciotto et al., 2012).

### Stress effects in β2^−/−^ mice

Stressed β2^−/−^ mice acquired C57BL/6J-like social behavior. That is, in β2^−/−^ mice, stress drastically reduced social contacts to the levels observed in C57BL/6J mice, and it normalized approaches, escapes and the number of stops. However, stress had no effect on tolerance, follows, or complex behaviors. After stress, follows were totally disconnected from all behavioral events (Figure 6). This disconnection may contribute to the reduced contact durations observed after stress. Sequences initiated by stop events were restricted, thus behavioral complexity was reduced (Figure 7). Finally, stress induced no additional dominance, but boosted aggressiveness. Individual data on chronograms and temporal evolution curves showed that the occurrence of aggression did not trigger systematic changes in social events; thus, these behavioral features were independent. In addition, emergence of aggressive behaviors seemed incompatible with elaboration of complex social sequences (Figure 7). Exploration was not associated to additional increase in plasma corticosterone in stressed β2^−/−^ mice. On the contrary, we observed a significant decrease in this stress marker at the end of the exploration period, as compared to the one measured immediately after immobilization. Thus, as for 57BL/6J mice, aggressiveness cannot be due to increases in plasma corticosterone amount. After stress, plasma corticosterone in β2^−/−^ mice tended to be higher (although this was not significant) than in C57BL/6J mice. This suggested that β2^−/−^ mice may be more sensitive to restraint stress (physical discomfort) than to stress generated by novelty exploration. Thus, stress would induce a focus on the internal state rather than on external stimuli. This focus on their internal state could produce attentional defects (Guillem et al., 2011) and trigger behavioral rigidity (Granon et al., 2003; de Chaumont et al., 2012). It was previously shown that elevation of catecholamines in the PFC inhibited prefrontal functional activity during stress (Arnsten, 2009) and that β2^−/−^ mice exhibited constitutive PFC noradrenaline elevation (Coura et al., 2013). A schematic of the catecholamine effect on PFC physiology exhibited a classical inverted U-shape (Arnsten, 2009); this relationship may underlie the inflexible social behavior observed in β2^−/−^ mice, as well as their even more inflexible behavior after stress.

In conclusion, stress in β2^−/−^ mice produced a dramatic reduction in the social repertoire, a clustering of behavioral sequences, emergence of aggressiveness, and apparent social normalization.

### Stress effects: similarities and differences in C57BL/6J and β2^−/−^ mice

C57BL/6J and β2^−/−^ mice exhibited similarly low baseline levels of corticosterone. Also, their levels of plasma corticosterone were comparable after immobilization stress and after stress followed by novelty exploration. This reinforced the idea that the behavioral differences reported here between stressed and not stressed C57BL/6J and β2^−/−^ mice could not be accounted for by plasma corticosterone absolute levels. However, variation in these levels differed in both genotypes: it remained stable whether animals explored novelty or were only stressed in C57BL/6J mice, whereas it significantly dropped after novelty exploration despite previous stress in β2^−/−^ mice. β2^−/−^ mice seemed to be more reactive to restraint stress than C57BL/6J mice. As mentioned above, this provided evidence that β2^−/−^ mice were more sensitive to their internal status than to external cues, leading, in consequences, to a larger variation in their corticosterone levels in between different behavioral conditions. The comparison between stressed C57BL/6J and stressed β2^−/−^ mice showed that acute stress tended to stereotype social actions of both mouse genotypes, and thus, it made both of them less flexible. This finding goes along with recent data obtained in humans, which showed that cold stress imposed before an instrumental task promoted habits (Schwabe and Wolf, 2009). As discussed above, this stress response also reminded behavior observed after NA PFC depletion, suggesting that acute stress may cause an imbalance in the NA levels in the PFC or, more generally, it may reflect impairment in PFC functions (Arnsten, 2009; Del Arco and Mora, 2009). This interpretation would be consistent with our previous findings that flexible social behaviors required functional nAChRs within the PFC (Avale et al., 2011), as they control the release of multiple ascending neurotransmitters in this brain region (dos Santos Coura and Granon, 2012).

## Conclusions

The present exhaustive behavioral analysis revealed that acute stress in adulthood was sufficient to trigger a marked increase in aggressiveness in both C57BL/6J and β2^−/−^ mouse genotypes. Because β2^−/−^ mice lacked nicotinic receptors, these results showed that aggressive bursts were not linked to nAChR function. We further showed that they were not linked to plasma corticosterone levels. In contrast, stress dramatically increased dominance only in C57BL/6J mice; this finding supports the notion that dominance and aggressiveness are unrelated processes, with only dominance depending on functional nAChRs. Acute stress impoverished mouse social interactions by altering the relationship between behavioral classes, and the classes altered depended on whether the mice exhibited a flexible or pathological social pattern. Thus, stress induced behavioral rigidity in “socially competent” animals, and it worsened rigid behavior in pathological models.

Taken together, these data showed that a unique experimental framework could tease apart behaviors in mice that represent components of the vicious stress-aggression cycle proposed in humans (Craig, 2007).

## Author contributions

Substantial contributions to the conception of the work: Sylvie Granon, Jean-Christophe Olivo-Marin, to the design of the work: Anne Nosjean, Sylvie Granon, to the acquisition analysis: Anne Nosjean, Arnaud Cressant, Fabrice de Chaumont, Frédéric Chauveau, to the interpretation of data: Anne Nosjean, Sylvie Granon, Arnaud Cressant, Fabrice de Chaumont, Frédéric Chauveau. Drafting the work: Anne Nosjean, Sylvie Granon; critical revision of the work: Anne Nosjean, Sylvie Granon, Arnaud Cressant, Fabrice de Chaumont, Frédéric Chauveau, Jean-Christophe Olivo-Marin. Final approval of the version to be published and agreement for all aspects of the work: Anne Nosjean, Sylvie Granon, Arnaud Cressant, Fabrice de Chaumont, Frédéric Chauveau, Jean-Christophe Olivo-Marin.

### Conflict of interest statement

The authors declare that the research was conducted in the absence of any commercial or financial relationships that could be construed as a potential conflict of interest.
