# Supplementary material for: Acute stress in adulthood impoverishes social choices and triggers aggressiveness in preclinical models
Source: Front Behav Neurosci. 2015 Jan 6;8:447. doi: 10.3389/fnbeh.2014.00447 (PMC4285129; doi:10.3389/fnbeh.2014.00447)
Supplement: Supplementary file 1 [file DataSheet1.DOCX]

**Supplementary Table 1. Social repertoire in the dyad of mice.** Categories and sub-categories of behavioral events during social interaction between an isolated host mouse (IH, black) and a social visitor mouse (SV, white) were choose using Mice Profiler software (de Chaumont et al., 2012). **(A)** Contact events (a1 to a6) rely on distance thresholds between the two mice of the dyad and do not take into account movements of the mice. We distinguished close contacts (a1 event) for which both mice were separated by 1cm or less, from other contacts (a2 to a6 events) for which mice were at whiskers distance (1.5 to 3cm apart). Within these latter contact events, we separated oral-oral, oral-genital, and side by side contacts. **(B)** Relative position events (b1, b2) defined the position of one mouse with regard to the other one (b1 and b2 events). These events did not include any distance or movement threshold. **(C-D)** Dynamic events initiated by the SV mouse (**C:** c1 to c4 events) or by the IH mouse (**D:** d1 to d6 events) take into account both distance and movement thresholds. They were divided in 3 categories: 1) first order events encompass distance between IH and SV mice, relative speed of one of the mice, and the direction of the movements, defining whether the mouse escapes, approaches or follows the other mouse (c1, c2, d1, d2 and d3 events), 2) complex second order events define sequences of 2 behaviors starting or finishing by a contact (c3, d4 and d5 events), 3) complex third order events embrace a succession of 3 events as approach-contact-escape, c4 and d6 events). (**E)** Stop events (e1, e2). SV and IH stop events were defined by mouse speed < 1.75 cm/s (e1 and e2 events). Stop of one mouse did not prejudge the behaviors of the other mouse. All the events were independently calculated and may thus arise simultaneously.

|  | | |  | Isolated Host mouse (IH, in black). |
| --- | --- | --- | --- | --- |
|  |  |  |  | Social Visitor mouse (SV, in white). |
| **A.** Contact Events | | a1 |  | IH and SV distance is < 1 cm. Mice are moving or not. |
|  |  | a2 |  | Oral-oral contact: distance between the centers of IH and SV heads is < 2 cm. Mice are moving or not. |
|  |  | a3 |  | Oral-genital contact (IH-SV): IH is behind SV at a distance < 1.5 cm. Mice are moving or not. |
|  |  | a4 |  | Oral-genital contact (SV-IH): SV is behind IH at a distance < 1.5 cm. Mice are moving or not. |
|  |  | a5 |  | Side by side (heads in the same direction): distances between both head and body of IH and SV are < 3 cm. Mice are moving or not. |
|  |  | a6 |  | Side by side (heads in opposite direction): distances between both head and body of IH and SV are < 3 cm. Mice are moving or not. |
| **B.** Relative position Events | | b1 |  | IH is behind SV (any distance), mice touching or not each other and moving or not. |
|  |  | b2 |  | SV is behind IH (any distance), mice touching or not each other and moving or not. |
| **C-D.** Dynamic Events | **C.** Events initiated by  SV mouse | c1 |  | SV escapes from IH (starting from no contact): SV’s speed > IH’s speed and the distance between mice increases over time. |
|  |  | c2 |  | SV approaches IH: SV’s speed > IH’s speed and the distance between mice decreases over time. If mice finally touch each other, this is event c3. |
|  |  | c3 |  | SV goes to and contacts IH (starting from no contact). |
|  |  | c4 |  | SV goes to and contacts IH which escapes. |
|  | **D.** Events initiated by IH mouse | d1 |  | IH escapes from SV (starting from no contact): IH’s speed > SV’s speed and the distance between mice increases over time. |
|  |  | d2 |  | IH approaches SV: IH’s speed > SV’s speed and the distance between mice decreases over time. If mice finally touch each other, this is event d4. |
|  |  | d3 |  | Follow behavior: IH is either behind or close on SV side at a distance < 1.5 cm with a speed >1.75 cm s^−1.^ |
|  |  | d4 |  | IH goes toward and contacts SV (starting from no contact). |
|  |  | d5 |  | SV escapes after contact with IH. |
|  |  | d6 |  | IH goes toward and contacts SV which escapes. |
| **E.**  Stop Events | | e1 |  | SV is immobile or exhibits a speed < 1.75 cm s^−1.^ |
|  |  | e2 |  | IH is immobile or exhibits a speed < 1.75 cm s^−1.^ |

**Supplementary Table 2. Individual mice aggressiveness during social interaction.** Signs – and + indicated absence and presence of aggressive events in each mice.

| **Mice** | **C57BL/6J Stress** | | **β2^-/-^ Stress** | |
| --- | --- | --- | --- | --- |
|  | Tail rattling | Attacks | Tail rattling | Attacks |
| **#1** | - | - | - | - |
| **#2** | + | - | - | - |
| **#3** | - | + | - | + |
| **#4** | + | + | + | + |
| **#5** | + | + | + | + |
| **#6** | + | + | + | + |
| **#7** | + | + | + | + |
| **#8** | + | + |  |  |

**Supplementary Figures Legends**

**Supplementary Figure 1. Experimental procedure.** IH mice (isolated for 4 weeks) were individually housed (in cage of the same dimension than the collective one) while SV mice remained group-housed. IH mice were C57BL/6J or β2^-/-^male mice, submitted or not to an acute stress. SV mice were always non stressed C57BL/6J male mice, approximately of same weight and age than IH mice. All behavioral experiments were performed from 9.00 a.m. to 2.00 p.m. One hour before the experiment, individually and group-housed mice were taken from the animal section and brought to a waiting room adjacent to the experimental room. Then, each isolated animal randomly assigned to the stressed groups was placed for 45 minutes in a Falcon® tube that was opened at the end to permit animals to breathe. The tube was taped at the bottom of an empty cage to prevent it from rolling. After this acute restraint stress period, each IH mouse was returned to its home cage for 5 minutes before being placed in the experimental box for 30 min exploration. Then, a SV mouse was gently introduced in the box, in the corner opposite to the IH mouse. Control mice were treated similarly, except that they were not submitted to stress. For corticosterone measures, others mice were sacrificed by decapitation. Blood samples were collected in different conditions. **Condition A**: in not stressed mice 60 min after exit from the animal facility (n= 4 C57BL/6J mice, n=4 β2^-/-^ mice); **Condition B**: just after stress (n= 6 C57BL/6J Stress mice, n=6 β2^-/-^ Stress mice) and **Condition C:** at the end of the exploration period (C57BL/6J mice: n=6 non stressed, n=7 stressed; β2^-/-^ mice: n=7 non stressed, n= 9 stressed).

**Supplementary Figure 2. Chronograms and density graphs of oral - genital and side by side contacts during social interaction.** Chronograms showed the temporal evolution of oral-genital (a3, a4) and side by side contact (a5, a6) events throughout the 4 minutes of the experiment for each dyad of IH - SV mice. Each line represents one dyad in a specific group. The length of each point is proportional to the duration of events. Graphs represented the mean of the temporal evolution of a given event for each group of mice. Vertical lines on chronograms and density graphs indicated the average latency to first attack in stressed C57BL/6J and stressed β2^-/-^ mice (full and dotted lines). C57BL/6J: blue, n = 11; C57BL/6J Stress: green, n = 8; β2^-/-^: yellow, n = 9; β2^-/-^ Stress: red, n = 7.

**Supplementary Figure 3. Chronograms and density graphs of four representative dynamic events during social interaction.** The chronograms illustrated the temporal evolution of dynamic events showed in **Figures 3 and 5**. Left (top and bottom): events initiated by SV mouse; c2: SV approaches IH; c4: SV approaches and contacts IH which escapes after contact. Right (top and bottom): events initiated by IH mouse; d2: IH approaches SV; d6: IH approaches and contacts SV which escapes after contact. Each line represents one dyad of IH - SV mice throughout the 4 minutes of the experiment in a specific group. Graphs below the chronograms represented the mean of the temporal evolution of the given event for each group of mice. Vertical lines on chronograms and density graphs indicated the average latency to first attack in stressed C57BL/6J and stressed β2^-/-^ mice (full and dotted lines). C57BL/6J: blue, n = 11; C57BL/6J Stress: green, n = 8; β2^-/-^: yellow, n = 9; β2^-/-^ Stress: red, n = 7.

**Supplementary Figure 4. Correlations between behavioral events during social interaction. (A)** Examples of correlation graphs. **(I)** Effect of stress in C57BL/6J mice: follow behavior was correlated to paw control in C57BL/6J (blue dots) and C57BL/6J stressed mice (green dots). **(II)** Effect of genotype: follow behavior was correlated to a5 event in C57BL/6J (blue dots) and β2^-/-^ mice (yellow dots). **(III)** Effect of stress in β2^-/-^ mice: a2 event was correlated to d1 behavior in β2^-/-^ (yellow dots) and β2^-/-^ stressed mice (red dots). *P* values are indicated. NS: not significant. See Supplementary Table S1 for behavioral symbols. Correlations were taken into account for *p* ≤ 0.0022. C57BL/6J: n = 11; β2^-/-^: n = 9; C57BL/6J Stress: n = 8; β2^-/-^ Stress: n = 7.
